# Identification of a major QTL and associated molecular marker for high arabinoxylan fibre in white wheat flour

**DOI:** 10.1371/journal.pone.0227826

**Published:** 2020-02-05

**Authors:** Alison Lovegrove, Luzie U. Wingen, Amy Plummer, Abigail Wood, Diana Passmore, Ondrej Kosik, Jackie Freeman, Rowan A. C. Mitchell, Kirsty Hassall, Mehmet Ulker, Karolina Tremmel-Bede, Marianna Rakszegi, Zoltán Bedő, Marie-Reine Perretant, Gilles Charmet, Caroline Pont, Jerome Salse, Michelle Leverington Waite, Simon Orford, Amanda Burridge, Till K. Pellny, Peter R. Shewry, Simon Griffiths

**Affiliations:** 1 Rothamsted Research, West Common, Herts, United Kingdom; 2 John Innes Centre, Norwich Research Park, Colney Lane, Norwich, United Kingdom; 3 Yuzuncu Yil University Faculty of Agriculture, Van, Turkey; 4 Centre for Agricultural Research, Agrártudományi Kutatóközpont, Martonvásár, Hungary; 5 INRAE UMR GDEC, 5 Chemin de Beaulieu, Clermont-Ferrand, France; 6 Life Sciences, University of Bristol, Bristol, United Kingdom; Institute of Genetics and Developmental Biology Chinese Academy of Sciences, CHINA

## Abstract

Dietary fibre (DF) has multiple health benefits and wheat grains are major sources of DF for human health. However, DF is depleted in white wheat flour which is more widely consumed than wholegrain. The major DF component in white flour is the cell wall polysaccharide arabinoxylan (AX). We have identified the Chinese wheat cultivar Yumai 34 as having unusually high contents of AX in both water-soluble and insoluble forms. We have therefore used populations generated from crosses between Yumai 34 and four other wheat cultivars, three with average contents of AX (Ukrainka, Altigo and Claire) and one also having unusually high AX (Valoris), in order to map QTLs for soluble AX (determined as relative viscosity of aqueous extracts of wholemeal flours) and total AX (determined by enzyme fingerprinting of white flour). A number of QTL were mapped, but most were only detected in one or two crosses. However, all four crosses showed strong QTLs for high RV/total AX on chromosome 1B, with Yumai 34 being the increasing parent, and a KASP marker for the Yumai 34 high AX allele was validated by analysis of high AX lines derived from Yumai 34 but selected by biochemical analysis. A QTL for RV was also mapped on chromosome 6B in Yumai 34 x Valoris, with Valoris being the increasing allele, which is consistent with the observation of transgressive segregation for this population. Association studies in an independent germplasm panel identified marker trait associations for relative viscosity in these same locations while direct selection for fibre content in breeding resulted in high levels of enrichment for the Yumai 34 1B allele. The data therefore indicate that marker-assisted breeding can be used to develop wheat with high AX fibre in white flour.

## Introduction

Dietary fibre (DF) is essential for human health, with cereals providing about 40% of the total fibre intake in Western European countries such as the UK [1] and Finland [2]. Dietary fibre (DF), and wholegrain cereal fibre in particular, has been shown to have a number of health benefits, including lowering blood pressure and serum cholesterol, improving insulin sensitivity and reducing the incidence of certain types of cancer, notably bowel and breast cancers [3]. The mechanisms for these benefits are still incompletely understood but are considered to include increasing faecal bulk and reducing intestinal transit time, binding cholesterol and carcinogens, reducing the rate of digestion and glucose release in the small intestine and fermentation to beneficial short chain fatty acids in the colon. DF also occurs in soluble and insoluble forms, which are considered to differ in some respects in their health benefits, with insoluble fibre being more slowly fermented and contributing particularly to binding cholesterol and carcinogens and increasing faecal bulk. However, despite these established health benefits, the intake of DF in most countries falls far below the recommended levels. For example, the daily intake in the UK is currently 17.2 g/day for women and 20.1 g/day for men, compared with a recommended intake of 30 g/day (https://www.nutrition.org.uk/nutritionscience/nutrients-food-and-ingredients/dietary-fibre.html).

Although wholegrain wheat is relatively rich in fibre, containing about 11 to 15% dry weight [4], most wheat products are made from white flour (derived from the starchy endosperm) [5] which contains only 2–3% fibre [6]. Furthermore, increased consumption of highly refined cereal products (including bread and other products from white flour) is occurring in countries undergoing urbanisation and industrialisation, which is considered to contribute to increases in obesity and chronic diseases in these countries [7].

The major components of the DF fraction of wheat flour are cell wall polysaccharides, principally arabinoxylan (AX) and (1→3,1→4)-β-D-glucan (β-glucan), which account for about 70% and 20% of the total cell wall polysaccharides, respectively, with about 2% cellulose ((1→4)-β-D-glucan) and 7% glucomannan [8]. However, the content of AX also varies between different genotypes of wheat. For example, 2-fold variation in the content of total (TOT)-AX and 4.7-fold variation in water-extractable (WE)-AX was reported in white flour of 150 wheat genotypes grown together on a single site [6], and 2.9-fold variation in WE-AX and 1.7-fold variation in water-unextractable (WU)-AX in 20 wheat cultivars [9]. Furthermore, a high proportion of the variation in the AX content of wholemeal and white flours of wheat is heritable, and hence accessible for exploitation by breeders [10,11]. However, the exploitation of this variation to develop improved wheats has been limited by the lack of tools for selection, with biochemical analyses being slow and costly and a lack of molecular markers for selection.

A number of studies of the genetic control of AX content have been reported, with most analysing wholemeal flour to determine AX (by monosaccharide analysis or colorimetric determination) or the relative viscosity of aqueous extracts as a proxy for AX. Martinant et al. [10] used two mapping populations comprising 91 doubled haploid (DH) lines from the cross Courtot x Chinese Spring and 115 single seed descent lines from the cross W7984 (synthetic) x Oparta. A major quantitative trait locus (QTL) was identified on chromosome 1B, which explained 32–37% of the variation in extract viscosity. Quraishi et al. [12] reported analyses of five additional crosses: 187 recombinant inbred lines (RILs) from the cross Courtot x Chinese Spring [13], 241 DH lines from the cross Arche x Recital [14], 194 RILs from the cross Renan x Recital [15], 124 DH lines from Valoris x Isengrain and 280 lines from RE006 x CF007 [16]. Several of these crosses had been analysed in previous studies and the data were therefore collated with new analyses to identify “meta-QTL”. In this way the 12 QTL identified in the five populations were reduced to three meta-QTL for WE-AX viscosity located on chromosomes 1B, 3D and 6B. The 1B QTL corresponded to that identified by Martinant et al. [10] while Charmet et al. [16] reported that the QTL on 6B accounted for up to 59% of the variation in WE-AX viscosity in the Valoris x Isengrain and RE006 x CF007 populations. More recently, Yang et al [17] reported a number of QTL for WE-AX (on chromosomes 1A, 1B, 2B, 3B, 5A, 5B, 7A and 7B) in a single population of 240 RILs from the cross PH82-2 x Neixiang 188 while Nguyen et al [18] identified major QTL for grain AX on chromosomes 2A and 4D and minor QTL on chromosomes 1A, 3D, 6B and 7A using a DH population of 154 lines from the cross Berku x Krichauff.

In addition, two association studies of AX in white flour of bread wheat and in wholemeal of tetraploid wheat have been reported. Quraishi et al. [12] complemented their meta-QTL analysis with association genetic analysis of the Healthgrain diversity collection of 156 wheat lines (131 winter and 20 spring bread wheats and five spelts) [6]. This identified seven loci involved in WE-AX viscosity: three co-located with the meta-QTL on chromosomes 1B, 3D and 6B and four additional loci on chromosomes 3A, 5B, 7A and 7B. Marcotuli et al [19] reported 19 QTL for AX content in a population of 104 tetraploid wheats.

However, although these studies identified a number of QTL, these were not consistent across crosses and have not led to the identification of markers for breeding. We have therefore determined the genetic control of AX content in white flour of wheat, by exploiting crosses with the high AX Chinese cultivar Yumai 34 [15] and through association genetics. Analysis of crosses between this cultivar and three cultivars with normal levels of fibre identified several QTLs, including a major QTL on chromosome 1B, while analysis of a cross with a second high AX genotype (Valoris) identified a second major QTL (on chromosome 6B). Association studies in an independent germplasm panel identified marker trait associations for relative viscosity in the same two locations. A linked marker for the 1B QTL was therefore developed and validated by analysis of high AX lines developed from Yumai 34 using biochemical analysis for selection.

## Materials and methods

### Production and growth of materials

Populations of Yumai 34 x Ukrainka (Y34Ukr), Yumai 34 x Claire (Y34Cla) and Yumai 34 x Valoris (Y34Val) were grown on three sites in the UK between 2012 and 2017: at Rothamsted Research (RR) (Hertfordshire, AL5, 2JQ), the John Innes Centre Church Farm (JI) (Norfolk, NR9 3PY and KWS UK Ltd. (Thriplow, Cambridgeshire, SG8, 7RE). At each field trial site three replicate 1m^2^ plots were grown per line in a randomised plot design. The populations Y34Val and Y34Alt were grown at INRA, Clermont Ferrand (FR), France in two replicates of 7.5m^2^ plots in 2013.

The cultivars Yumai 34, Ukrainka and Lupus were also grown in replicated field trials in Martonvásár (Hungary) from 2009–2018 and high fibre AX selected from a cross between Yumai 34 and Ukrainka as described in [20].

### Milling

White flour was produced using a Chopin CD1 mill. Grains were brought to room temperature and moisture content determined using Bruker Minispec mq-20 NMR analyser using an in-house developed calibration. 50g of grain was conditioned to 16.5% moisture overnight prior to milling. First break and first reduction flours were combined to give the white flour fraction.

Wholemeal flour was produced from mature grain of Y34Ukr and Y34Cla using a Retch centrifugal mill with a 250μm sieve. Wholemeal flours of Y34Alt and Y34Val were produced by ball milling at 8°C with a 500μm sieve. All wholemeal and white flour samples were immediately stored at -20°C prior to use.

### Determination of relative viscosity (RV)

RV of aqueous extracts of white flours and of wholemeal flours of Y34Ukr and Y34Cla was determined as described dx.doi.org/10.17504/protocols.io.babqiamw [21]. Relative viscosities of wholemeal flours of Y34Alt and Y34Val were determined at 30°C using an automated viscometer (AVS 310, CV (%) 23 22 8 Schott Gerate, Germany) fitted with an Ostwald capillary (2 mL, 0·4 mm) [22].

### Arabinoxylan analysis

Total (TOT) and water-extractable (WE)-AX in white flour were determined by monosaccharide analysis, by GC-FID of alditol acetates as described by Gebruers et al [23] using the method of Englyst et al [24]. Results were adjusted for the presence of arabinogalactan as described in Gebruers et al [23]. TOT and WE)-AX were also determined in white flour samples as pentosan as described by Finnie et al [25] based on Douglas [26].

Enzymatic fingerprinting of AX was as described dx.doi.org/10.17504/protocols.io.babriam6

[27]. At least two technical replicates of each biological replicate were analysed. The areas under the arabinoxylan oligosaccharide (AXOS) peaks were combined to determine TOT-AX (expressed in arbitrary units).

### Markers and mapping

If not stated differently, genetic mapping, plotting and QTL mapping was conducted in the R software suite (vs. 3.6.1) [28]. Genetic maps were constructed using the Axiom® 35K breeders’ array genotypes, scored by the Functional Genomics Group at the University of Bristol. Of the 35143 markers only those polymorphic in the respective populations were used. These were for Y34Alt, Y34Cla, Y34Ukr and Y34Val: 5514, 3368, 3569, and 5647 markers, respectively. A custom script was developed to conduct the genetic mapping for Y34Cla, Y34Ukr and Y34Val employing package ASMap (vs. 1.0–4), using default settings with the exception of “p.value” = 10^−12^ or 10^−13^ (Y34Val), “noMap.dist” = 20, “noMap.size” = 2, “miss.thresh” = 10. In a second mapping round, it was attempted to join linkage groups from the same chromosome together, using setting “p-value" = 10^−3^ or 10^−1^ (Y34Val). As the calculated map units seemed to be inflated, each map was corrected to a total length of 2100 cM. The Y34Alt map was constructed using MSTmap online (http://mstmap.org/) with default setting and a grouping threshold of LOD = 10. Pictures of the genetic maps were plotted using package “LinkageMapView” (vs. 2.1.2) (S1 Fig). QTL detection was performed using package “qtl” (vs. 1.44–9) [29] in two steps, the first scan determining co-factors and the second scan identifying robust QTL, taking the co-factors into account [30]. The QTL analysis was performed for the traits RV and TOT-AX from six trials (Y34Alt + Y34Val (only RV): FR 2013, Y34Ukr: RH 2013, Y34Cla: JI 2015 + 2016, Y34Val (only TOT-AX): RH2017) employing the genetic maps. In total, 21 significant QTL were identified, of which 12 had a LOD score of three or over (S1 Table and S2 Fig.). New KASP markers were developed, where necessary, from SNPs detected by Axiom markers using assays shown on CerealsDB (https://www.cerealsdb.uk.net/cerealgenomics/CerealsDB/) predesigned using polymarker [31]. The KASP primers used for SNP BA00789946 (shown as AX-94524314 on the genetic maps presented here) were: acaactaactatgcaagtgcca (FAM labelled), acaactaactatgcaagtgccg (VIC labelled) and ggatgacacatctcaagaaaaagaa. Standard conditions for the KASP PCR were used, with a hydrocycler and a touchdown of 65°C to 57 ^O^C.

### Association analysis

The whealbi panel (www.whealbi.eu) was used for genome wide association genetics in order to confirm the QTLs found in biparental populations. This panel is described in Pont et al [32] and comprised 435 bread wheat genotypes. RV was measured on wholemeal flour of grain of 426 lines harvested in Martovasar, Hungary, in 2015. Exome capture and NGS sequencing were achieved, leading to 620,158 robust genetic variants in wheat (among them 595,939 SNPs and InDels between hexaploid accessions) distributed across 41,032 genes. These biallelic markers were used with the GWAS function of R library rrBLUP [32]. To avoid false positives, a mixed model was used, with kinship estimated with all markers except those located on the chromosome on which markers are tested for association. A FDR of 5% was used to account for multiple correlated tests.

## Results

### Identification of the Chinese wheat cultivar Yumai 34 as a source of high arabinoxylan fibre in white flour

Analysis of 150 bread wheat genotypes in the EU Healthgrain project identified the Chinese wheat cultivar Yumai 34 as having the highest contents of both water-extractable (WE) AX and total (TOT) AX in white flour, with 1.4% water-extractable WE-AX and 2.75% TOT-AX, compared with mean values for the 150 lines of 0.51% and 1.93%, respectively [6]. The high proportion of WE-AX was especially notable, corresponding to over 50% of TOT-AX in Yumai 34 compared with a mean of 26% for the 150 lines.

Although these analyses were carried out on grain samples from single plots grown on the same site in 2005–6, further comparative analyses carried out on lines grown in the UK and Hungary have confirmed that Yumai 34 always contains the highest, or among the highest, contents of both TOT and WE- AX fractions in flour. In particular, comparison of three genotypes grown in Hungary for 10 seasons showed that Yumai 34 contained a mean of 9.52 mg AX/g dry wt. (range 8.43–11.41) compared to 5.68 (range 4.35–6.93) for Ukrainka and 6.52 (range 5.42–7.28) for Lupus (S2 Table).

### Development of populations for genetic analysis of AX

Four populations were generated from crosses between Yumai 34 and European cultivars: the central European bread making cultivar Ukrainka (96 F6 RILs) (Y34Ukr), the UK biscuit-making cultivar Claire (95 RILs) (Y34Cl) and the French bread making cultivars Altigo (245 DHLs) (Y34Alt) and Valoris (84 DHLs) (Y34Val). Whereas Ukrainka, Altigo and Claire and were selected as parents because they have average contents of AX, previous studies have shown that Valoris has higher than average contents of both WE-AX and TOT-AX (0.8% and 2.2% compared with means of 0.51% and 1.93%, respectively, in the Healthgrain study) and it has therefore been used as a “high AX parent” in crosses with low AX genotypes [16].

### Mapping relative viscosity (RV) of aqueous extracts of wholemeal flour

Most previous genetic analyses of AX fibre in wheat have determined the RV of aqueous extracts of white or wholemeal flours as a proxy for arabinoxylan content [11,12]. This is because RV is more readily determined than AX, eliminating the need for milling to produce white flour and biochemical analysis. It is considered to be valid because WE-AX is known to be the major contributor to the viscosity of aqueous extracts. However, the use of RV of wholemeal flour as a proxy for WE-AX in white flour has not been validated. We therefore compared the RV of aqueous extracts and the contents of WE-AX in wholemeal and white flours from 10 lines from the Y34Ukr cross (grown at Rothamsted in 2013/4). This showed good correlations between wholemeal RV and wholemeal WE-AX (determined as pentosans) (r = 0.70), white flour RV (r = 0.84) and white flour WE-AX (pentosans) (r = 0.77) (S3 Fig.)

**Fig 1:**
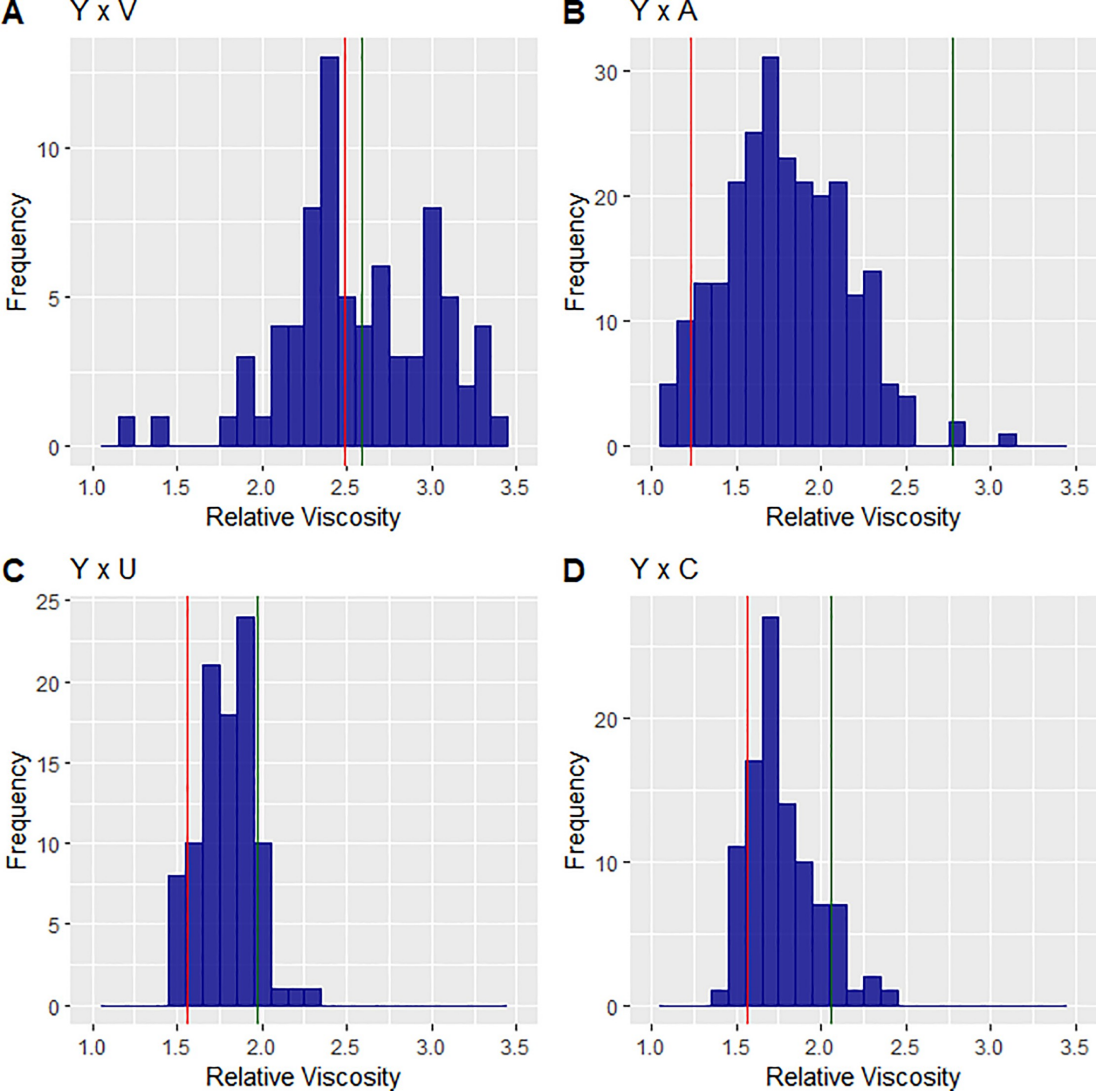
Relative viscosity of wholemeal flours. Wholemeal flours from DH populations from Y34Val (A), Y34Alt (B), Y34Ukr (C) and Y34Cla (D). Parental lines are shown in green (Yumai 34) and red (Valoris, Altigo, Ukrainka, Claire).

The RV of aqueous extracts from single samples of wholemeal flours from the four populations was therefore determined. This showed clear transgressive segregation in the population derived from the cross with Valoris (which has high AX) (Fig 1A), but not in the populations derived from crosses between Yumai 34 and the lines with average AX: Altigo, (Fig 1B), Ukrainka (Fig 1C) and Claire (Fig 1D).

**Fig 2:**
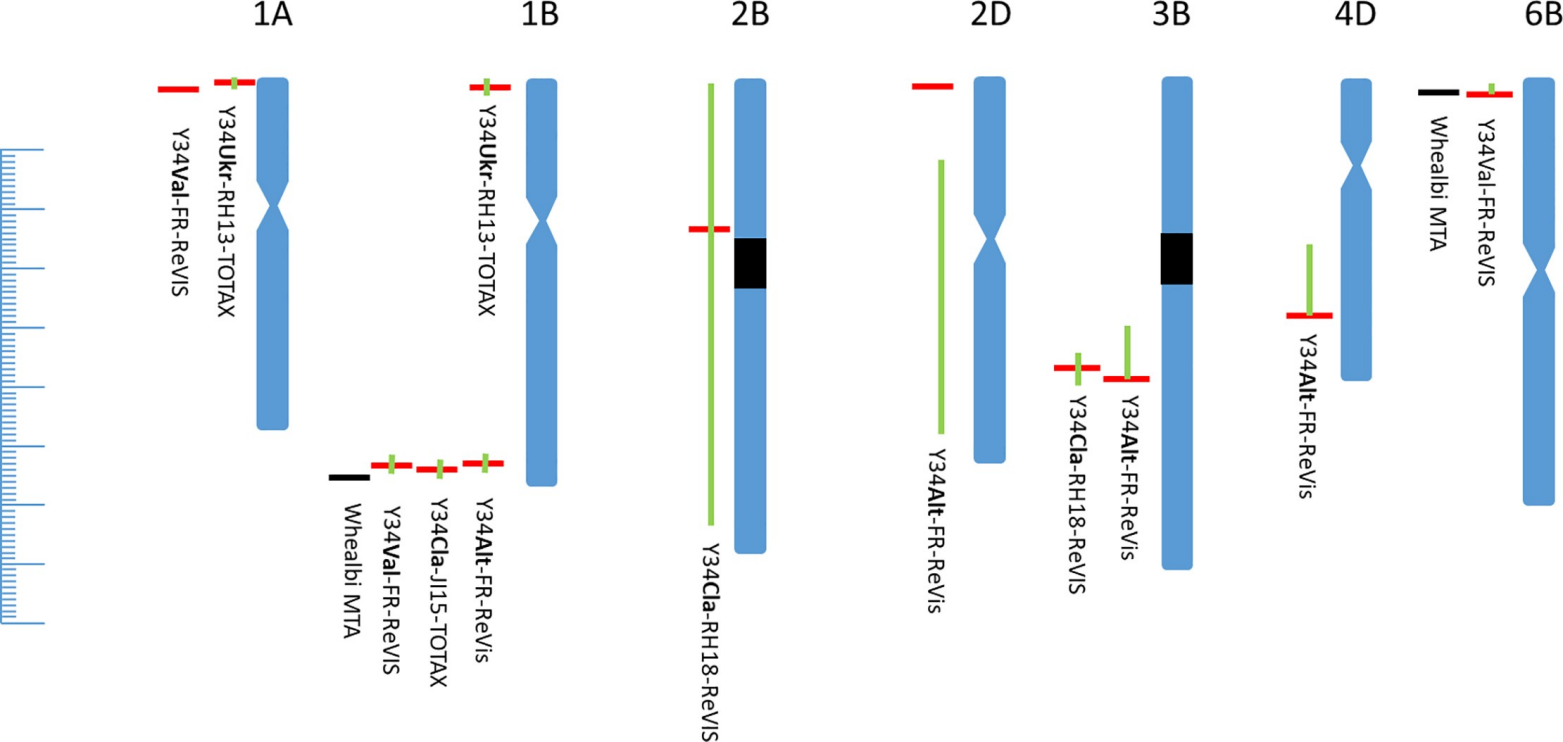
Chromosomal locations of genetic effects for RV and TOT-AX mapped in the four crosses. Alignment of QTL peak marker (horizontal red bar) and flanking markers defining 1 LOD confidence interval (edges of green vertical bar) is to Chinese RefSeq v1.0. Each hash mark on the left-hand scale represents 1Mb. Approximate locations of centromeres are shown as an hour glass when determined using chromosome arm survey sequence data [34] and as black blocks for 2B and 3B using density of annotated genes [33]. QTL are named as population-environment-trait (see Table 1).

The QTLs with LOD score >3 identified in these crosses are shown in Table 1. Seven RV QTLs were identified on chromosomes 1AS, 1BL, 2BS, 2D, 3BL, 4DL, and 6BS: four in Y34Alt, 2 in Y34Cl, none in Y34Ukr and 3 in Y34Val. These QTL positions are projected onto the genome of Chinese Spring [33] in Fig 2. The relative mapped positions of flanking and peak markers for the 2D QTL may suggest rearrangement. Where the QTL 1 LOD confidence intervals substantially overlap (green line on figure) for the same trait in different populations we have made the assumption that the underlying effect is the same. The increasing alleles for these QTL come from Yumai 34 except for a QTL for RV on 1A in Y34Val and a QTL for RV on 6B in Y34Val.

**Table 1:** QTLs above LOD threshold 3 for RV and TOT-AX in four crosses.

Population	Chr	Env (location and year)	Trait	LOD	%var	add eff	mean	increasing Allele
Y34Alt	1B	FR13	RV	12.6	16.3	-0.53	3.0	Y34
	2D	FR13	RV	3.2	3.7	-0.26	3.0	Y34
	3B	FR13	RV	6.1	7.4	-0.3	3.0	Y34
	4D	FR13	RV	3.5	4.1	-0.27	3.0	Y34
Y34Cla	2B	JI16	RV	3.1	11.4	-0.038	1.8	Y34
	3B	JI16	RV	5.9	23.9	-0.013	1.8	Y34
	1B	JI15	TOT-AX	3.2	14.1	-3.8	74.4	Y34
Y34Ukr	1A	RR13	TOT-AX	3.2	13.0	2.1	95.1	Ukr
	1B	RR13	TOT-AX	5.1	21.6	-3.0	95.1	Y34
Y34Val	1A	FR13	RV	3.8	10.3	0.62	4.5	Val
	1B	FR13	RV	7.8	24.2	-0.99	4.5	Y34
	6B	FR13	RV	4.4	12.3	0.85	4.5	Val

Y34, Yumai 34; Alt, Altigo; Cla, Claire; Ukr, Ukrainka; Val, Valoris. Chr is the chromosome on which QTL are identified; env is the environment in which the QTL was detected (location and year): FR, Clermont Ferrand, JI, John Innes Centre, RR, Rothamsted Research; % var is the percentage of phenotypic variance explained; mean is the mean of the trait for the whole population, increasing allele indicates the direction of the allelic effect.

A major objective of this study was to understand the genetic basis of the high RV of Yumai 34, and in particular to identify increasing alleles of QTLs which have large effects and are expressed in multiple populations. In this respect, the 1BL QTL stands out at LOD 12.6 and 7.8 in the Y34Alt and Y34Val populations, respectively, accounting for 16.3% and 24.2% phenotypic variance. With additive effects of 0.53 and 0.99 RV units this QTL alone can deliver substitution effects of 1 to 2 RV units in populations with mean RVs of 3 and 4.5. A weaker RV effect was also detected on chromosome 3B in two populations, Y34Alt and Y34Cla. No other RV effects were detected in more than one population.

### Mapping TOT-AX determined by fingerprinting

Analyses of ten Y34Ukr lines (discussed above) showed that RV of aqueous extracts of wholemeal was a poor predictor of TOT-AX (calculated as the sum of the AXOS released) in white flour (r = 0.38) (S3 Fig). We therefore determined the amount of TOT-AX by enzyme fingerprinting of 3 replicate samples of the Y34Ukr, Y34Cl and Y34Val populations.

Three TOT-AX QTL were identified in the Y34Ukr and Y34Cl populations. Both populations segregated for a TOT-AX QTL on chromosome 1B with Yumai 34 carrying the increasing allele. However, alignment to the wheat genome sequence (Fig 2) shows that the locations on 1B are very different, sub-telomeric 1BL in Y34Cl and sub-telomeric 1BS in Y34Ukr. For the third TOT-AX QTL Ukrainka carries the increasing allele on 1AS.

### Coincidence of RV and TOT-AX QTL

Fig 2 shows how some of the QTL for the two traits that were mapped in the four populations co-locate. The Claire TOT-AX QTL on 1BL co-locates with the QTL for RV from Y34Alt and Y34Val, with Yumai 34 carrying the increasing allele in all cases. Two further co-locating QTL are not shown in Table 1 or Fig 2 because the LOD scores were below the cut-off. These were a QTL for TOT-AX in Y34Val (LOD 2.5) and a QTL for RV in Y34Ukr (LOD2.3).

QTLs for RV with Yumai 34 carrying the increasing allele were co-located on chromosome 3B in Y34Alt and Y34Cl and were also identified on chromosomes 2B (Y34Alt), 4D (Y34Alt) and 2B (Y34Cla).

Three QTL were identified in which Yumai 34 was not the increasing parent, for TOT-AX and RV from Y34Ukr and Y34Val, respectively, which co-located on chromosome 1A, and for RV on chromosome 6B in Y34Val only.

### Identification and validation of markers for the high AX QTL from Yumai 34

The QTLs identified on 1BL for which Yumai 34 alleles increased TOT-AX and/or RV were prioritised for the development of KASP SNP assays which would allow validation of the QTL effects in independent populations and the selection of high AX Yumai 34 alleles in breeding programmes. The five Axiom markers nearest to the RV QTL peak of Y34Alt were therefore selected for KASP marker development. The SNP BA00789946 detected by AX-94524314 was converted into a robust KASP assay and used to genotype the Y34Alt population, giving exactly the same segregation pattern as the corresponding Axiom marker.

**Fig 3:**
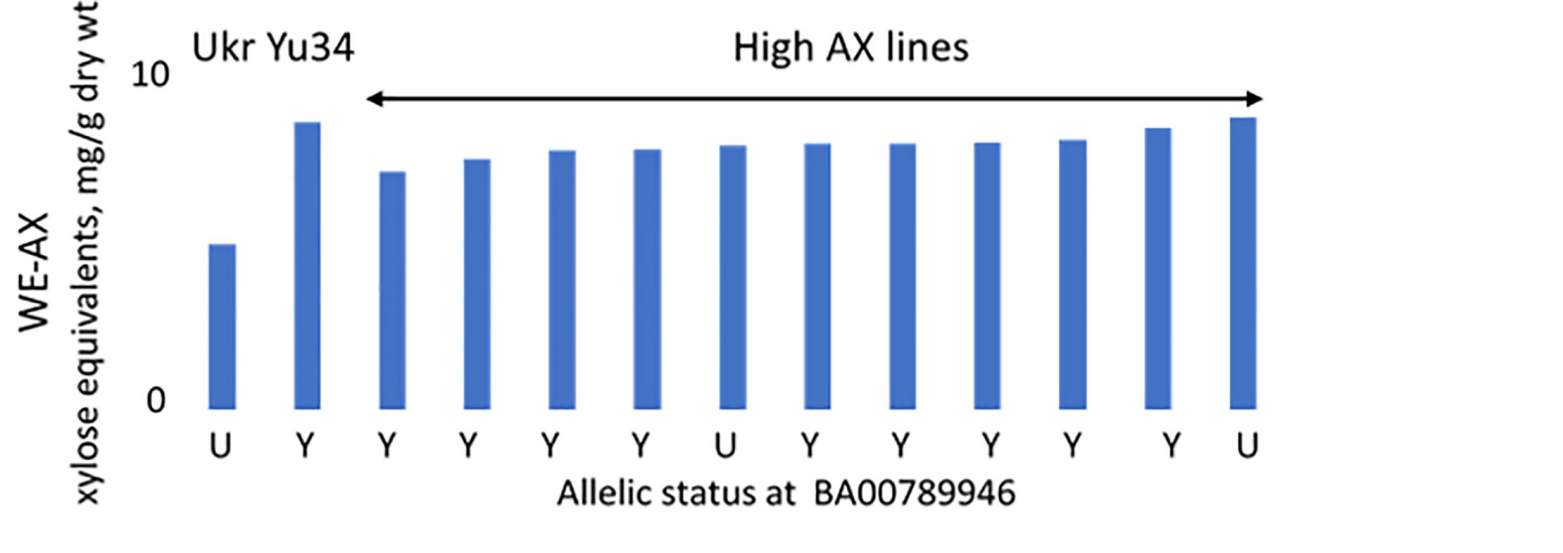
Contents of WE-AX in white flours and allelic status at the 1BL QTL determined using marker BA00789946. AX is determined as pentosan (expressed as xylose equivalents) in white flours of 11 high AX lines derived from the cross Yumai 34 x Ukrainka using biochemical analysis for selection. Data are the means of samples grown at Martonvasar over 3 years (2013–2015) as previously reported [20].

The marker was then validated using high AX breeding lines developed by Tremmel-Bede et al. [20] from crosses between Yumai 34 and conventional cultivars. These lines were selected by determining the contents of WE-AX and TOT-AX in white flour, showing increases in WE-AX of about 0.5% and in TOT-AX of about 1% compared to the conventional parents. Of eleven high fibre lines selected from a cross between Yumai 34 and Ukrainka, nine carried the Yumai 34 allele of the BA00789946 KASP marker. The alleles in these lines are shown together with the contents of WE-AX in white flour in Fig 3. A second set of selections from the same study was genotyped from a cross between Yumai 34 and the German cultivar Lupus. Although no polymorphism was detected using DNA from available accessions of Yumai 34 and Lupus, with both carrying the Yumai34 allele of BA00789946, the nine high AX progeny did segregate with eight carrying the Yumai 34 allele and one the alternative allele.

### Validation by GWAS analysis

Further validation was provided by GWAS analysis of the WHEALBI collection (www.whealbi.eu) using a mixed model with kinship estimated with markers from all chromosomes (S3 Table). This showed marker association on the two chromosomes on which signals above the FDR5% threshold were detected: chromosomes 1B and 6B (Fig 2). These marker trait associations have -log p values >10 and discrete significance peaks. In each case, the increasing allele was not rare, 80 and 150 out of 426 for the 1B and 6B, alleles, respectively. These results validate the strongest QTLs found in the bi-parental populations.

## Discussion

We have used four crosses with the high fibre wheat cultivar Yumai 34 to identify QTL for high RV and total AX fibre in white flour. Although a number of QTL were mapped, which is consistent with earlier studies, most were only detected in one or two crosses. However, all four crosses showed strong QTLs for high AX/RV on chromosome 1B, with Yumai 34 being the increasing parent, although this mapped to the 1BS in the Y34Ukr and to 1BL in the other crosses. Furthermore, a KASP marker for the high AX Yumai 34 allele was validated by analysis of high AX lines derived from Yumai 34 but selected by conventional biochemical analysis.

Previous studies have also shown QTLs for RV on chromosome 1B, including a QTL on 1BL with a heritability of 27.4% in one population [16]. This may correspond to the 1BL QTL in Yumai 34, with the Yumai 34 allele giving higher levels of AX and RV. The two QTL on 1B described here appear to be genuinely independent. In support of this, the BA00789946 KASP assay is assigned to 1BL when mapped using the Y34Ukr population, as in the other three crosses. Nevertheless, this marker has strong predictive power for high fibre in the progeny selected from crosses between Yumai 34 and Ukrainka, which supports the QTL for RV on 1BL in this population even though the LOD score was below the cut-off.

The most likely explanation for the identification of a TOT-AX QTL on 1BS in the cross with Ukrainka is that Yumai 34 does indeed carry AX increasing effects at both ends of chromosome 1B. However, detecting linked QTLs requires higher statistical power and it is likely that we did not detect the TOT-AX QTL on 1BL as significant in the same cross due to insufficient power.

It is notable that only one cross, between Yumai 34 and the high AX cultivar Valoris, showed significant transgressive segregation. Previous analysis of a cross between Valoris and Isengrain (which has a normal AX level) identified a QTL on 6BL which explained 58.4% of the heritability [12, 16]. This may correspond to the QTL identified on 6B in the Yumai 34 x Valoris cross, although this QTL was mapped to 6BS not 6BL. This QTL was also found using GWAS in the Whealbi panel of 426 bread wheat lines, with the Yumai allele having a negative effect on viscosity. The presence of the high AX allele of the 1B QTL in Valoris probably accounted for the presence of transgressive segregation, which indicates that it should be possible to stack the 1B and 6B QTLs, and possibly also other high AX QTLs.

## Supporting information

S1 Fig Pictures of the genetic maps of Y34Alt, Y34Cla, Y34Ukr and Y34Val. The centiMorgan scale is at the left side of each figure. Linkage groups are depicted as vertical bars (light blue). Marker positions are given as horizontal lines across the linkage groups and the names of the markers are on the right side of each linkage groups. For co-segregating markers only one marker name is given.
(PDF)

S2 Fig Plots of the QTLscans for traits RV and TOT-AX in the four populations. Only plots with QTL LOD scores above the significance threshold are shown. LOD scores (left) are plotted against chromosome map positions in cM. Marker names are given along the chromosome axis, markers in black are border marker of the confidence interval and markers in red are QTL peak markers. Horizontal lines at the bottom of the plot show the extend of the CI. Abbreviations: Allelic effects are given in the legend (top left): A (-) increasing effect on Y34, B(+) increasing effect on the second parent of the cross.
(PDF)

S3 Fig Correlation Matrix of 10 lines from the Yumai 34 x Ukrainka population grown at Rothamsted Research in 2013–2014. WE-AX and TOT-AX were measured colorometrically (pentosan assay) and TOT-AX by enzyme fingerprinting in wholemeal and white flour.
(PDF)

S1 Table Summary table of QTL identified using traits RV and TOT-AX including significant QTL with LOD scores under 3.0 (Minor QTL). Abbreviations: Y34 Yumai 34; Alt Altigo; Cla Claire; Ukr Ukrainka; Val Valoris; chr chromosome on which QTL were identified; env: environment (location and year): FR: Clermont Ferrand, JI: John Innes Centre, RH: Rothamsted Research; %var percentage of phenotypic variance explained; increasAll: increasing allele (A = Y34, B = second parent); LG chr: linkage group on the genetic map; cM: centiMorgan; CI confidence interval of the QTL; RefSeq1.0 Chr: chromosome on the IWGCS Reference Sequence (vs 1.0) whole genome assembly; pos: positions on the chromosome, if given in base pairs (bp) positions refer to RefSeq1.0.
(PDF)

S2 Table Comparison of the contents of total and water-extractable AX (determined as pentosan) in white flour from three wheat cultivars grown in field trials over 10 years (2009–2018). (PDF)

S3 Table Marker trait associations identified for relative viscosity in the WHEALBI panel. (PDF)
