# Supplementary figures and images for: Identification of a major QTL and associated molecular marker for high arabinoxylan fibre in white wheat flour
Source: PLoS One. 2020 Feb 5;15(2):e0227826. doi: 10.1371/journal.pone.0227826 (PMC7001892; doi:10.1371/journal.pone.0227826)

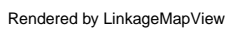

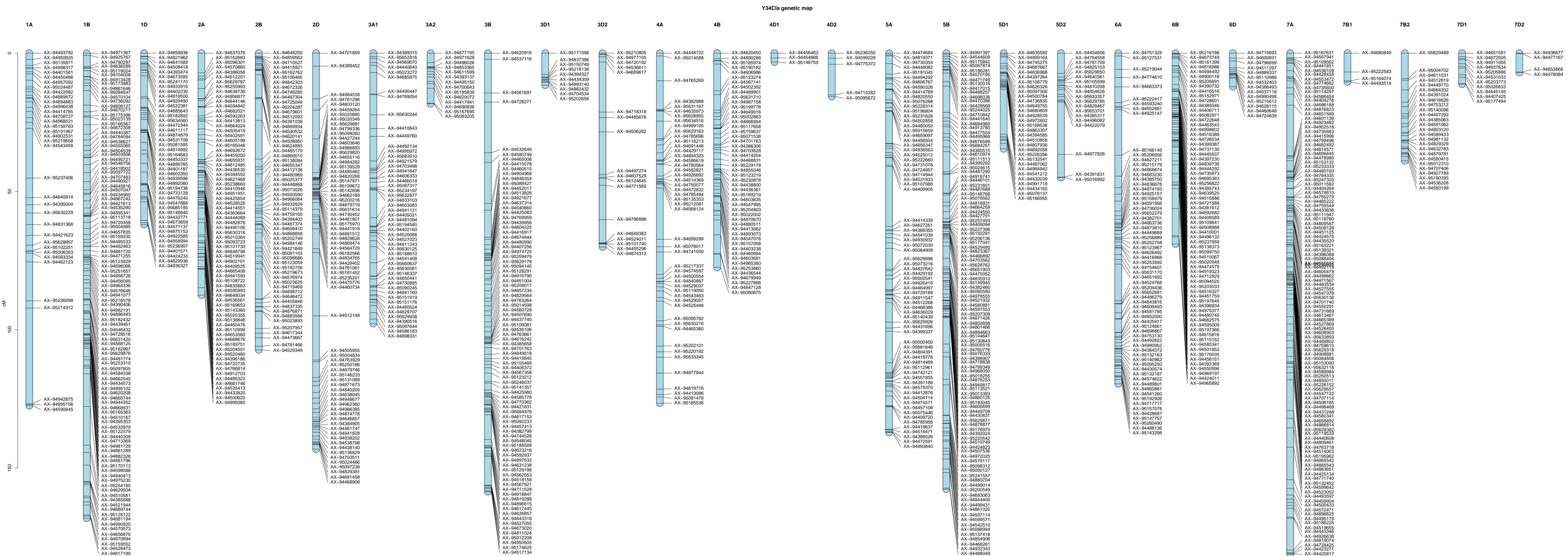



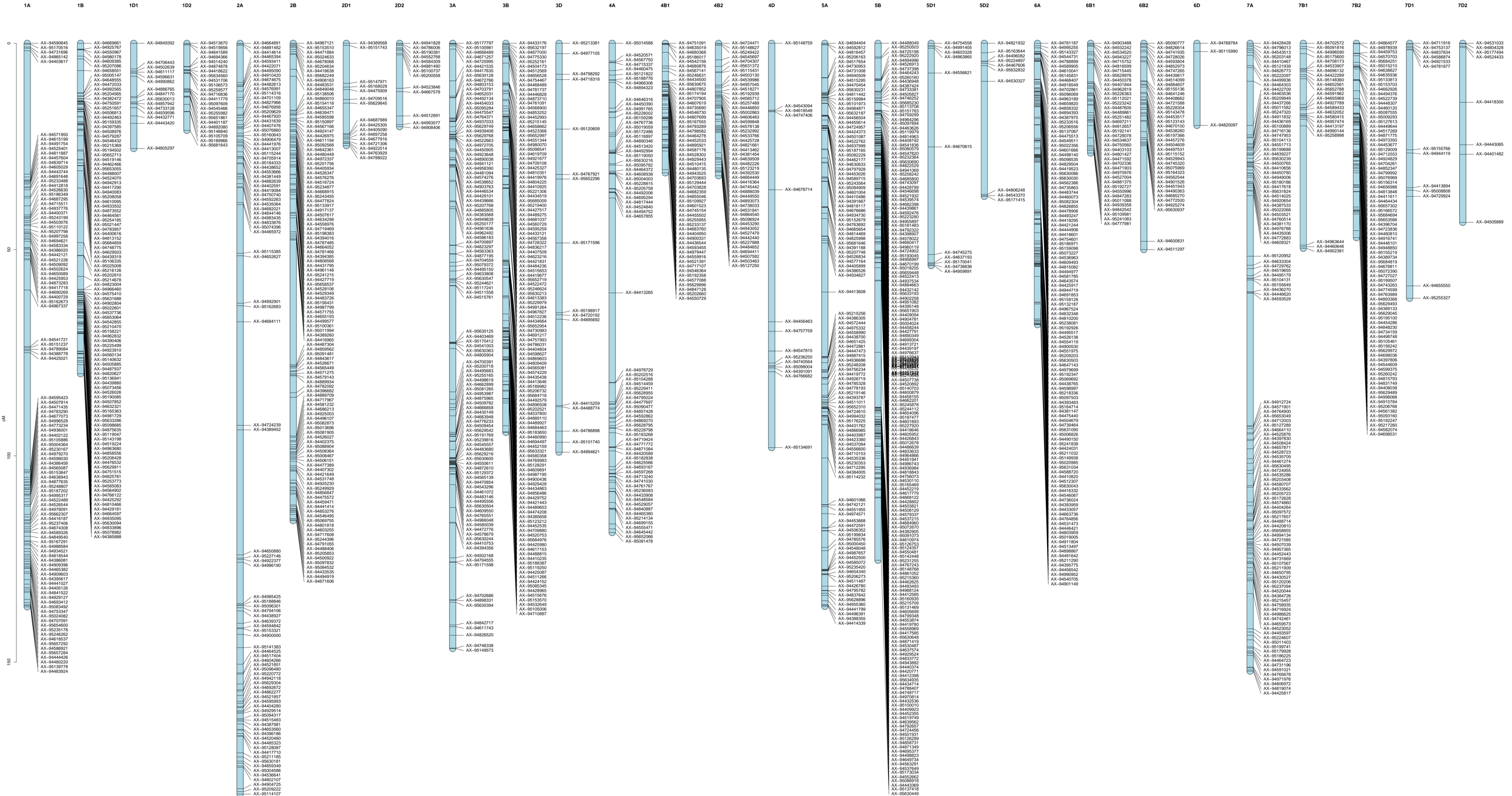

Supplement: S1 Fig — The centiMorgan scale is at the left side of each figure. Linkage groups are depicted as vertical bars (light blue). Marker positions are given as horizontal lines across the linkage groups and the names of the markers are on the right side of each linkage groups. For co-segregating markers only one marker name is given. (PDF) [file pone.0227826.s001.pdf]
