# Supplementary material for: Identification of a major QTL and associated molecular marker for high arabinoxylan fibre in white wheat flour
Source: PLoS One. 2020 Feb 5;15(2):e0227826. doi: 10.1371/journal.pone.0227826 (PMC7001892; doi:10.1371/journal.pone.0227826)
Supplement: S2 Fig — Only plots with QTL LOD scores above the significance threshold are shown. LOD scores (left) are plotted against chromosome map positions in cM. Marker names are given along the chromosome axis, markers in black are border marker of the confidence interval and markers in red are QTL peak markers. Horizontal lines at the bottom of the plot show the extend of the CI. Abbreviations: Allelic effects are given in the legend (top left): A (-) increasing effect on Y34, B(+) increasing effect on the second parent of the cross. (PDF) [file pone.0227826.s002.pdf]

QTLs: Y34Alt − ReVis − 1B

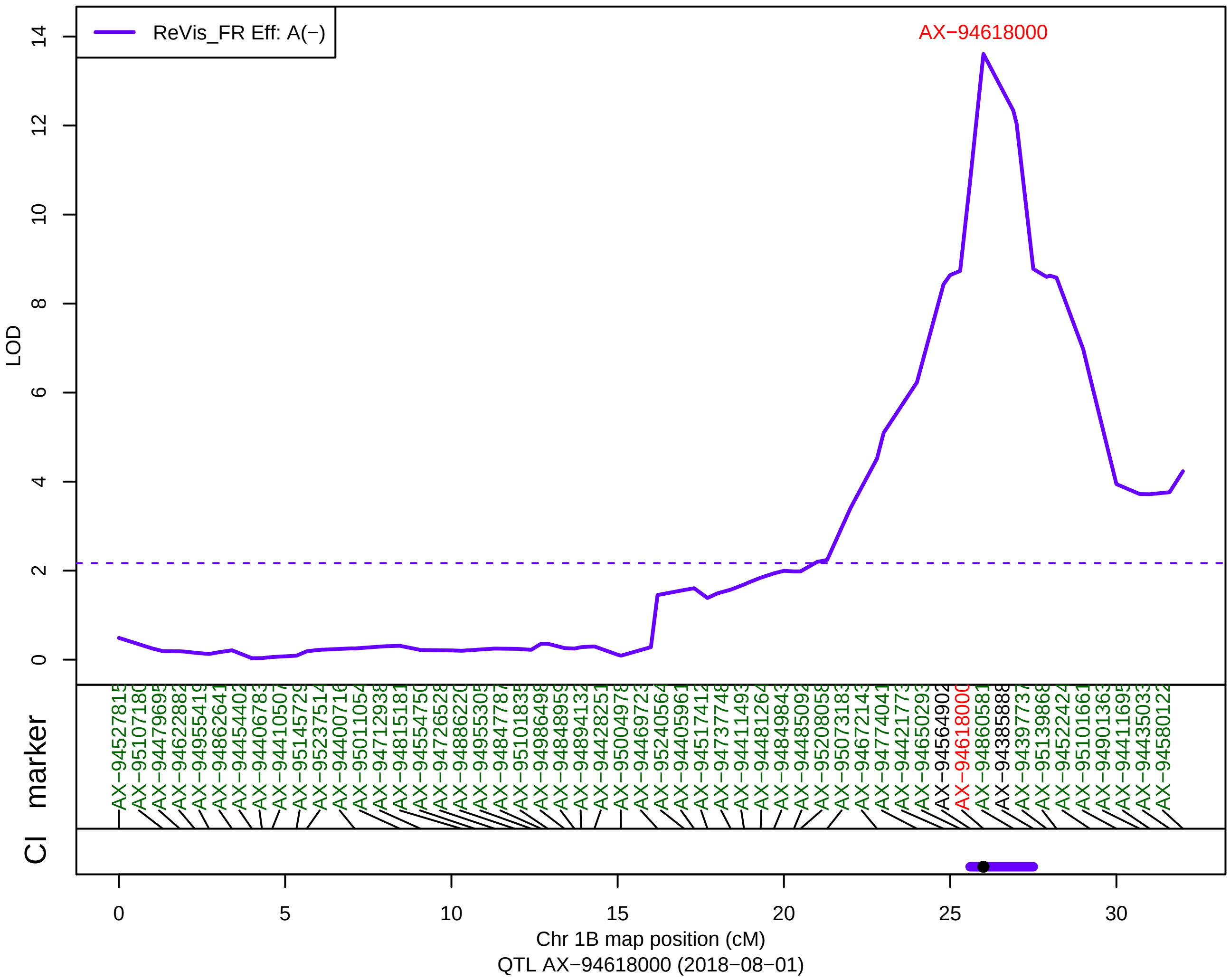

QTLs: Y34Alt − ReVis − 2B

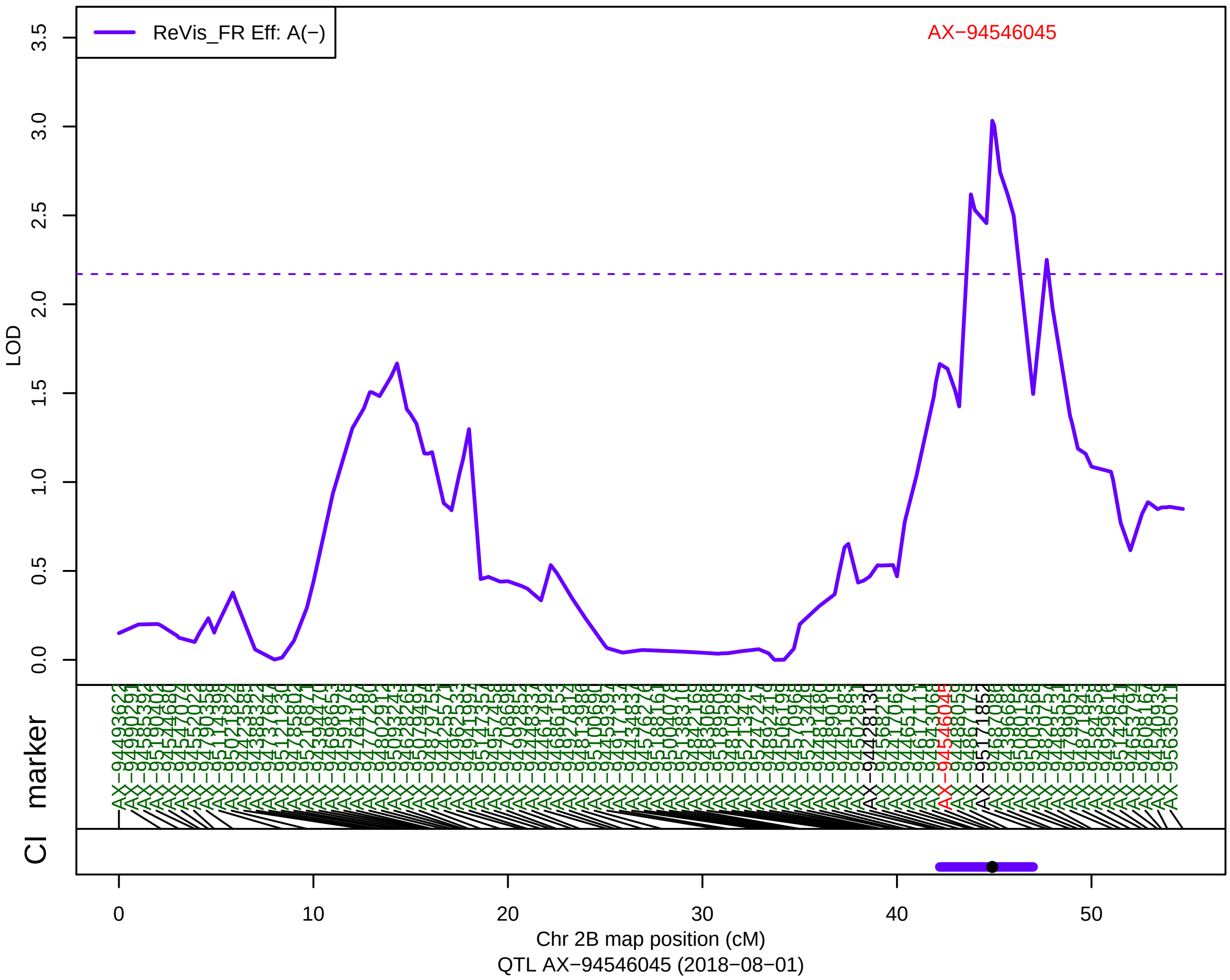

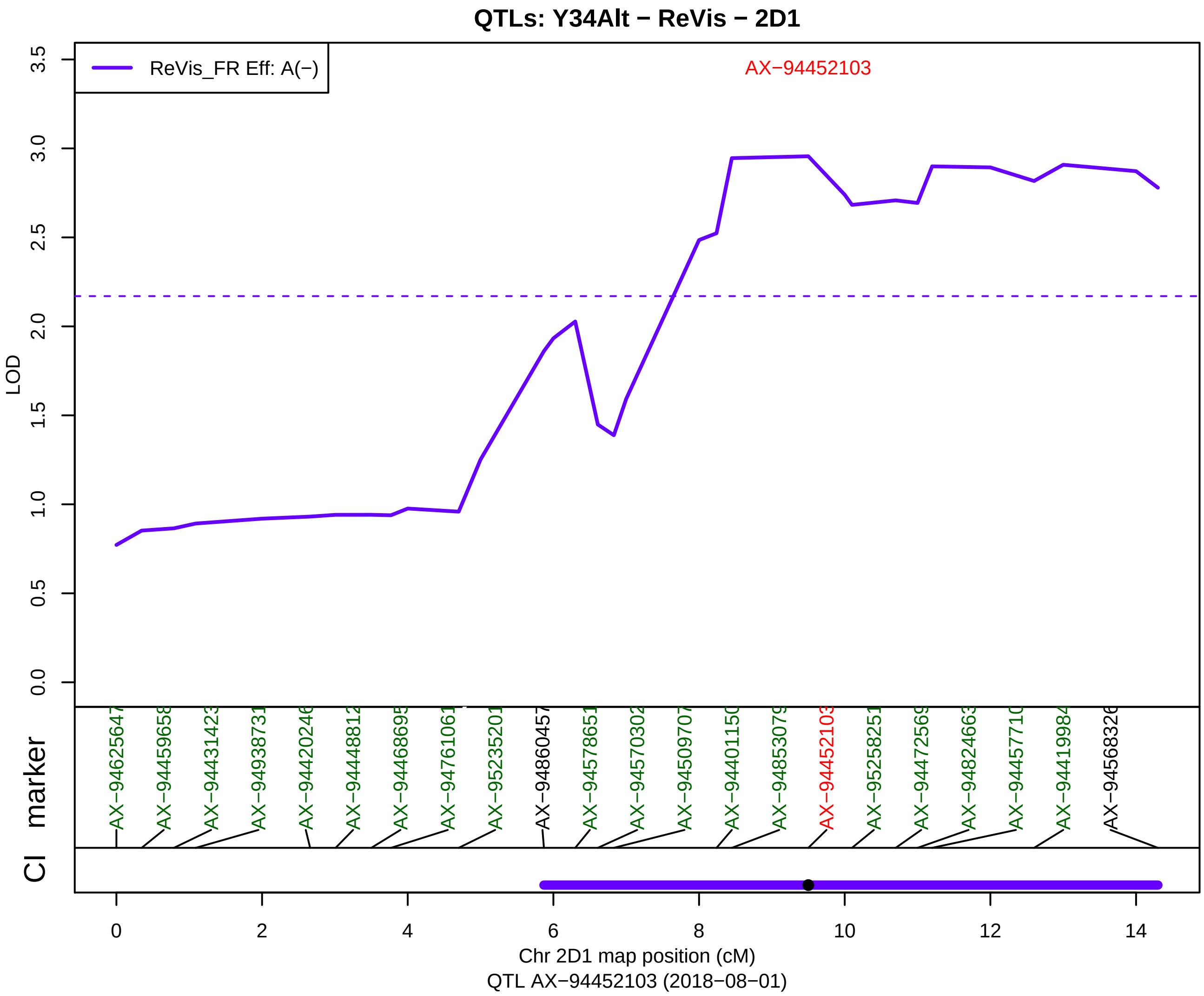
QTLs: Y34Alt − ReVis − 2D1
ReVis_FR Eff: A(−)
AX−94452103
LOD
0.0
0.5
1.0
1.5
2.0
2.5
3.0
3.5
marker
CI
AX−94625647
AX−94459658
AX−94431423
AX−94938731
AX−94420246
AX−94448812
AX−94468695
AX−94761061
AX−95235201
AX−94860457
AX−94578651
AX−94570302
AX−94509707
AX−94401150
AX−94853079
AX−94452103
AX−95258251
AX−94472569
AX−94824663
AX−94457710
AX−94419984
AX−94568326
0
2
4
6
8
10
12
14
Chr 2D1 map position (cM)
QTL AX−94452103 (2018−08−01)

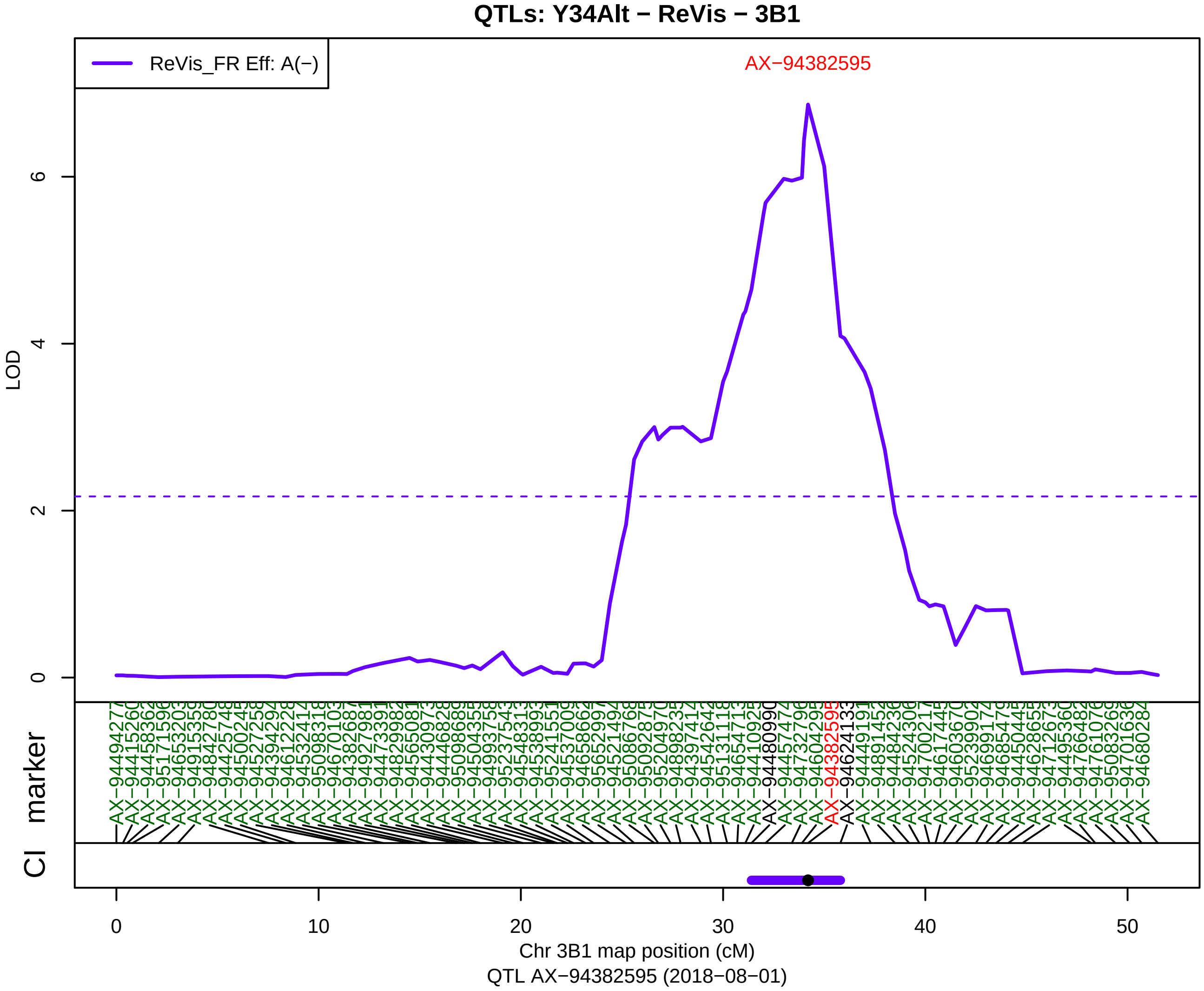
QTLs: Y34Alt − ReVis − 3B1
ReVis_FR Eff: A(−)
AX−94382595
LOD
0
2
4
6
marker
CI
0
10
20
30
40
50
Chr 3B1 map position (cM)
QTL AX−94382595 (2018−08−01)

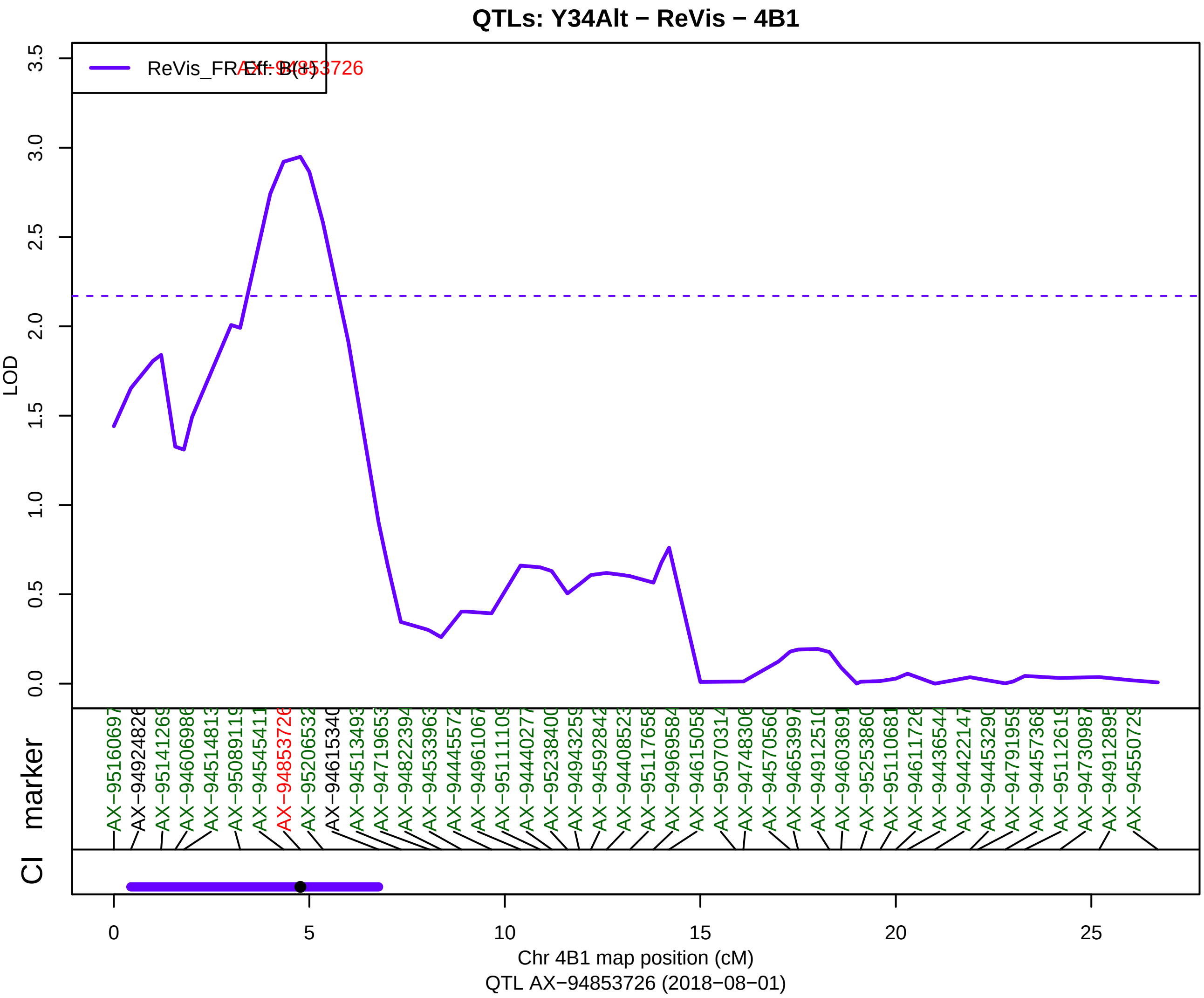
QTLs: Y34Alt − ReVis − 4B1
ReVis_FR Eff: B(+)
AX−94853726
LOD
0.0
0.5
1.0
1.5
2.0
2.5
3.0
3.5
marker
CI
AX−95160697
AX−94924826
AX−95141269
AX−94606986
AX−94514813
AX−95089119
AX−94545411
AX−94853726
AX−95206532
AX−94615340
AX−94513493
AX−94719653
AX−94822394
AX−94533963
AX−94445572
AX−94961067
AX−95111109
AX−94440277
AX−95238400
AX−94943259
AX−94592842
AX−94408523
AX−95117658
AX−94969584
AX−94615058
AX−95070314
AX−94748306
AX−94570560
AX−94653997
AX−94912510
AX−94603691
AX−95253860
AX−95110681
AX−94611726
AX−94436544
AX−94422147
AX−94453290
AX−94791959
AX−94457368
AX−95112619
AX−94730987
AX−94912895
AX−94550729
0
5
10
15
20
25
Chr 4B1 map position (cM)
QTL AX−94853726 (2018−08−01)

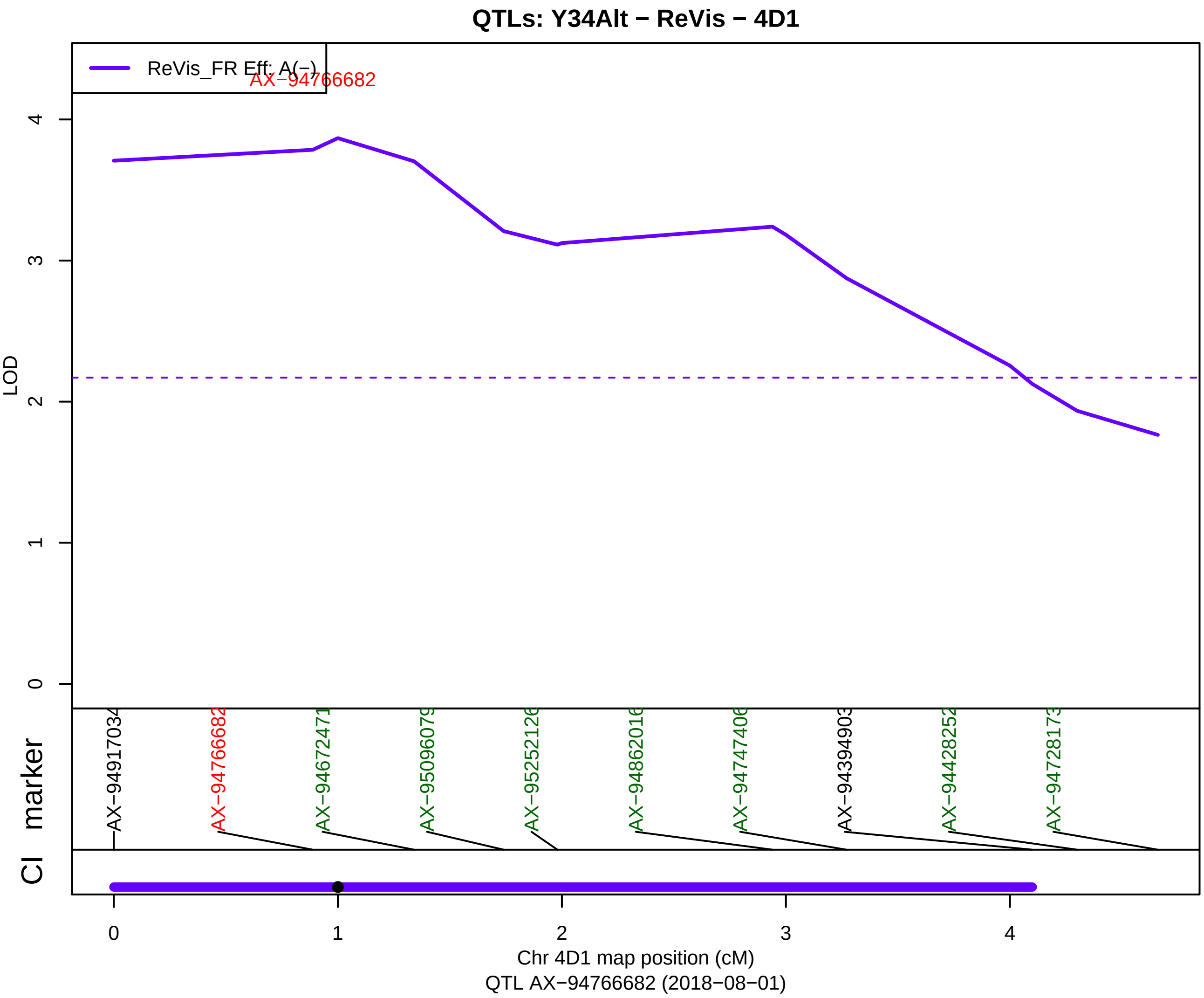
QTLs: Y34Alt − ReVis − 4D1
ReVis_FR Eff: A(−)
AX−94766682
LOD
0
1
2
3
4
marker
CI
AX−94917034
AX−94766682
AX−94672471
AX−95096079
AX−95252126
AX−94862016
AX−94747406
AX−94394903
AX−94428252
AX−94728173
0
1
2
3
4
Chr 4D1 map position (cM)
QTL AX−94766682 (2018−08−01)

QTLs: Y34Cla − TOTAX − 1B

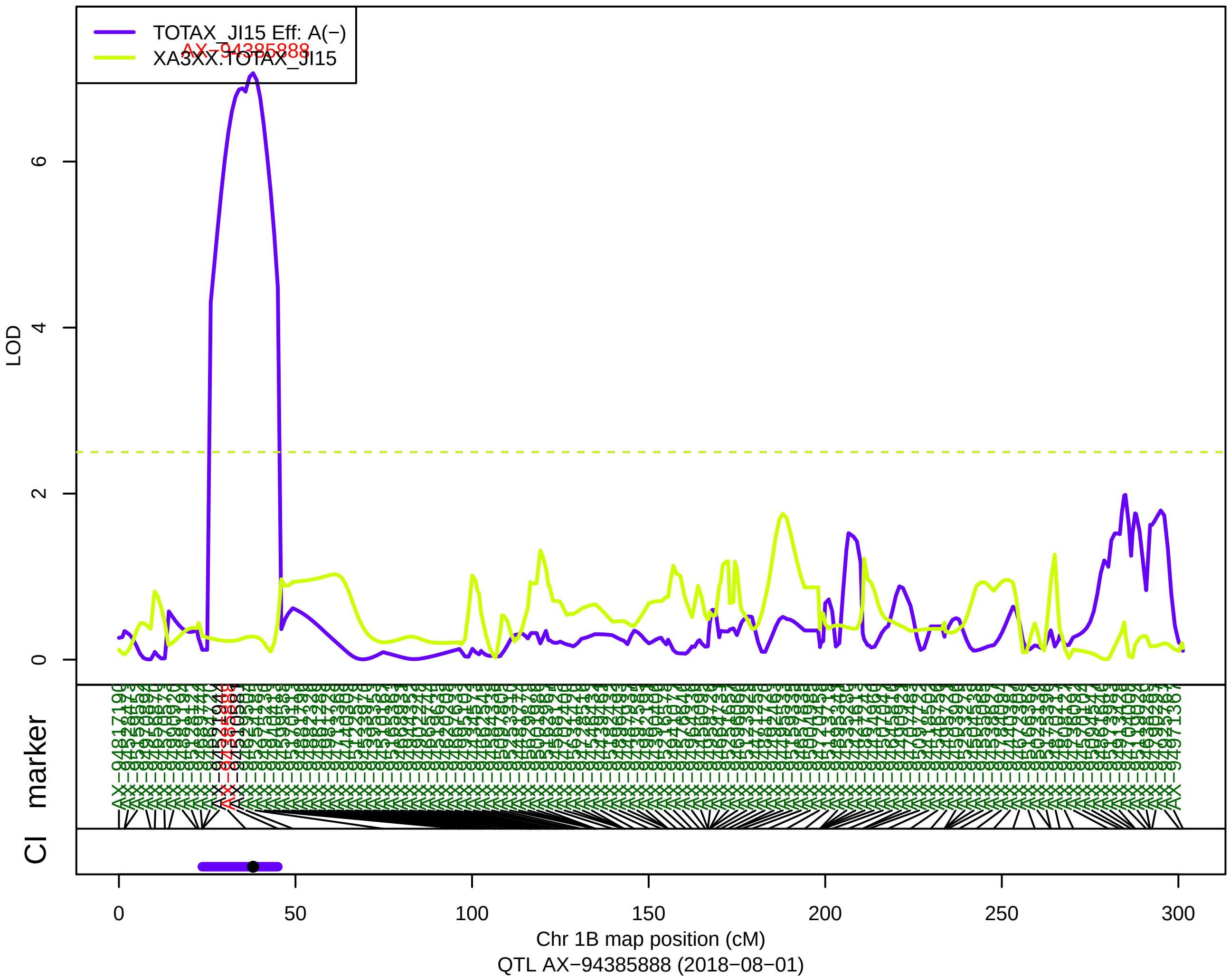

QTLs: Y34Cla − TOTAX − 5D2

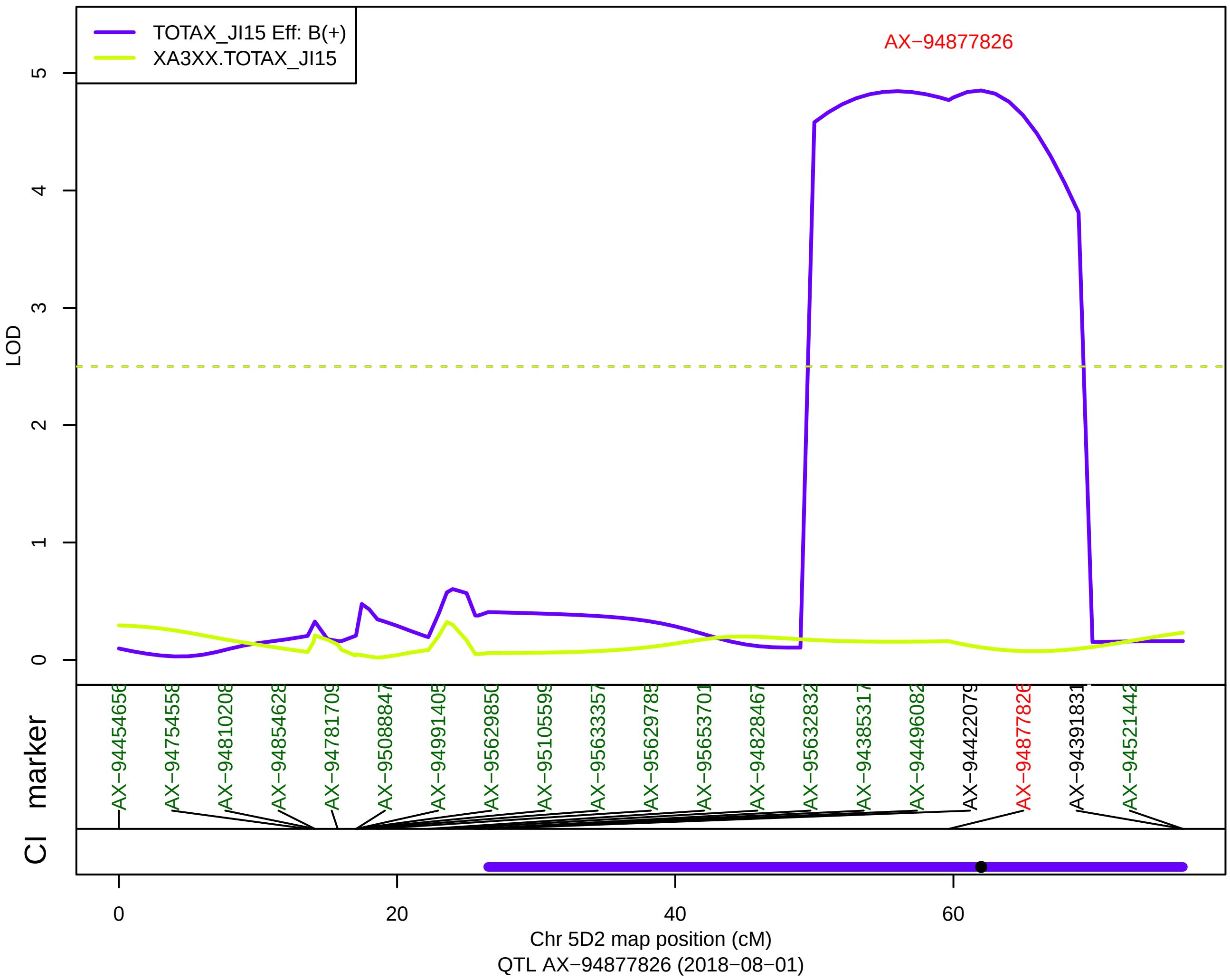

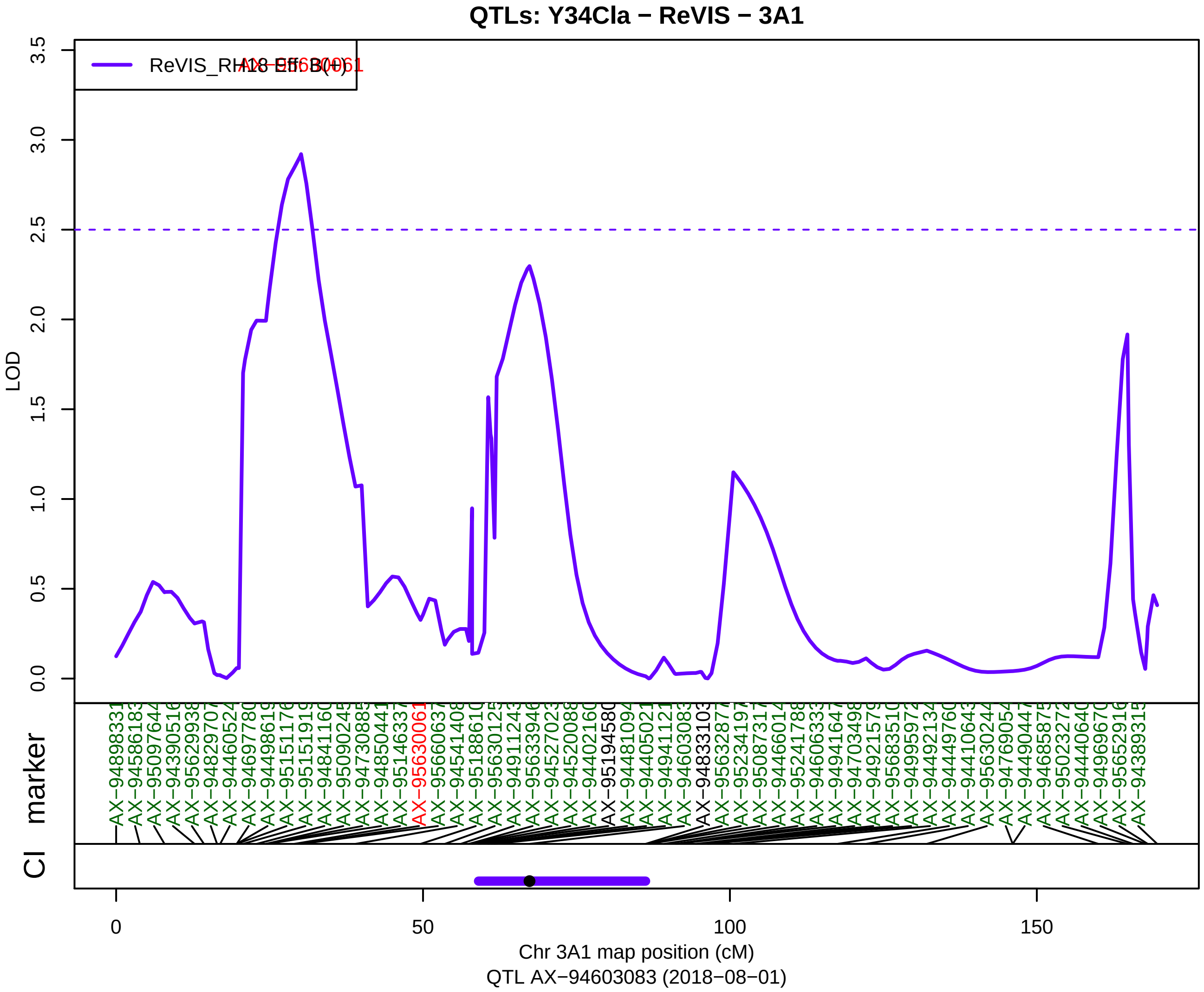
QTLs: Y34Cla − ReVIS − 3A1
ReVIS_RH18 Eff B(+)
AX−95630061
LOD
0.0
0.5
1.0
1.5
2.0
2.5
3.0
3.5
marker
CI
AX−94898331
AX−94586183
AX−95097644
AX−94390516
AX−95629938
AX−94829707
AX−94460524
AX−94697780
AX−94498619
AX−95151176
AX−95151919
AX−94841160
AX−95090245
AX−94730885
AX−94850441
AX−95146337
AX−95630061
AX−95660637
AX−94541408
AX−95188610
AX−95630125
AX−94911243
AX−95633946
AX−94527023
AX−94520088
AX−94402160
AX−95194580
AX−94481094
AX−94405021
AX−94941121
AX−94603083
AX−94833103
AX−95632877
AX−95234197
AX−95087317
AX−94466014
AX−95241788
AX−94606333
AX−94941647
AX−94703498
AX−94921579
AX−95683510
AX−94995972
AX−94492134
AX−94449760
AX−94410643
AX−95630244
AX−94769054
AX−94490447
AX−94685875
AX−95023272
AX−94440640
AX−94969670
AX−95652916
AX−94389315
0
50
100
150
Chr 3A1 map position (cM)
QTL AX−94603083 (2018−08−01)

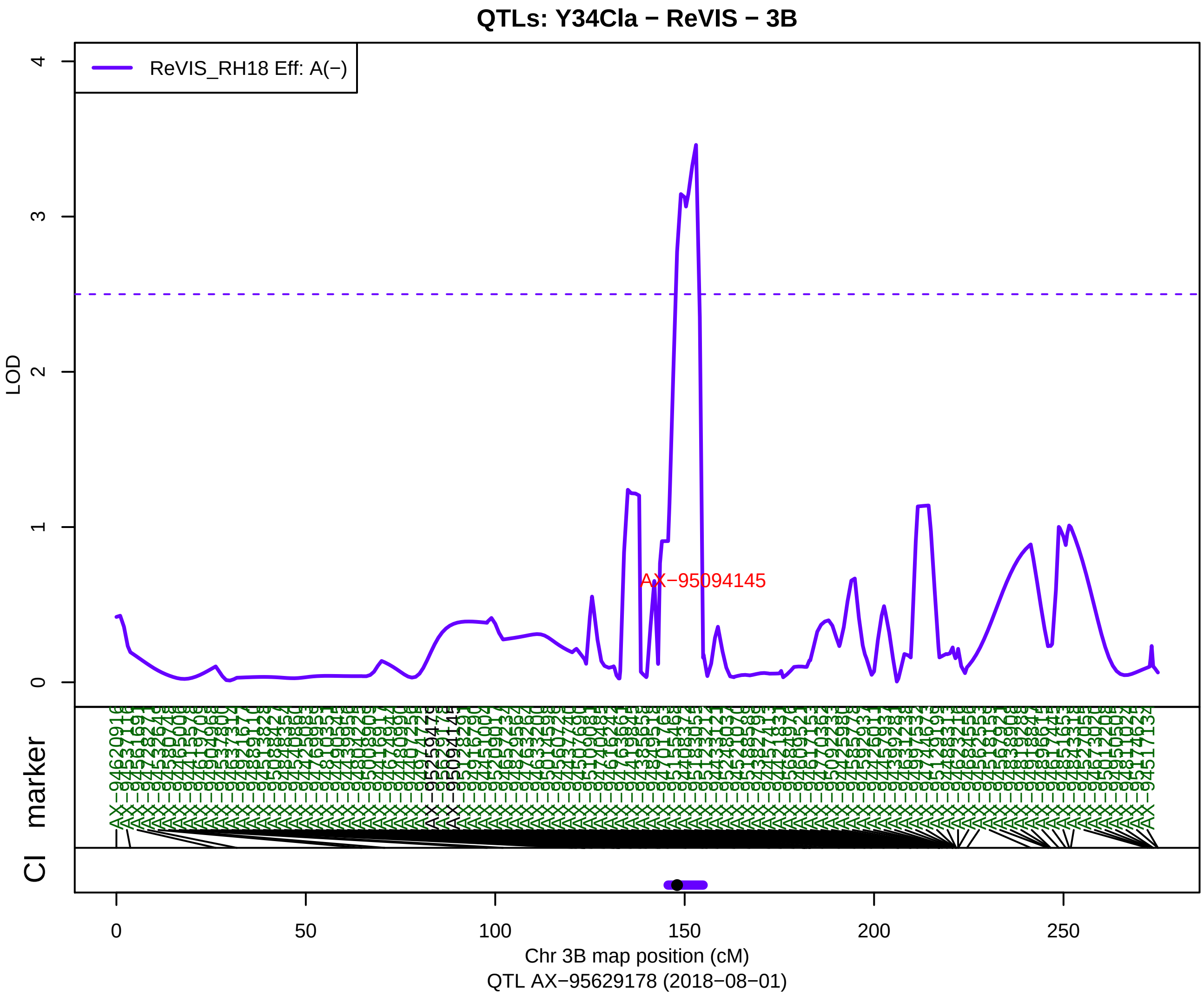

QTLs: Y34Cla − ReVIS − 3B
ReVIS_RH18 Eff: A(−)
LOD
AX−95094145
marker
CI
Chr 3B map position (cM)
QTL AX−95629178 (2018−08−01)

QTLs: Y34Cla − TOTAX − 2D

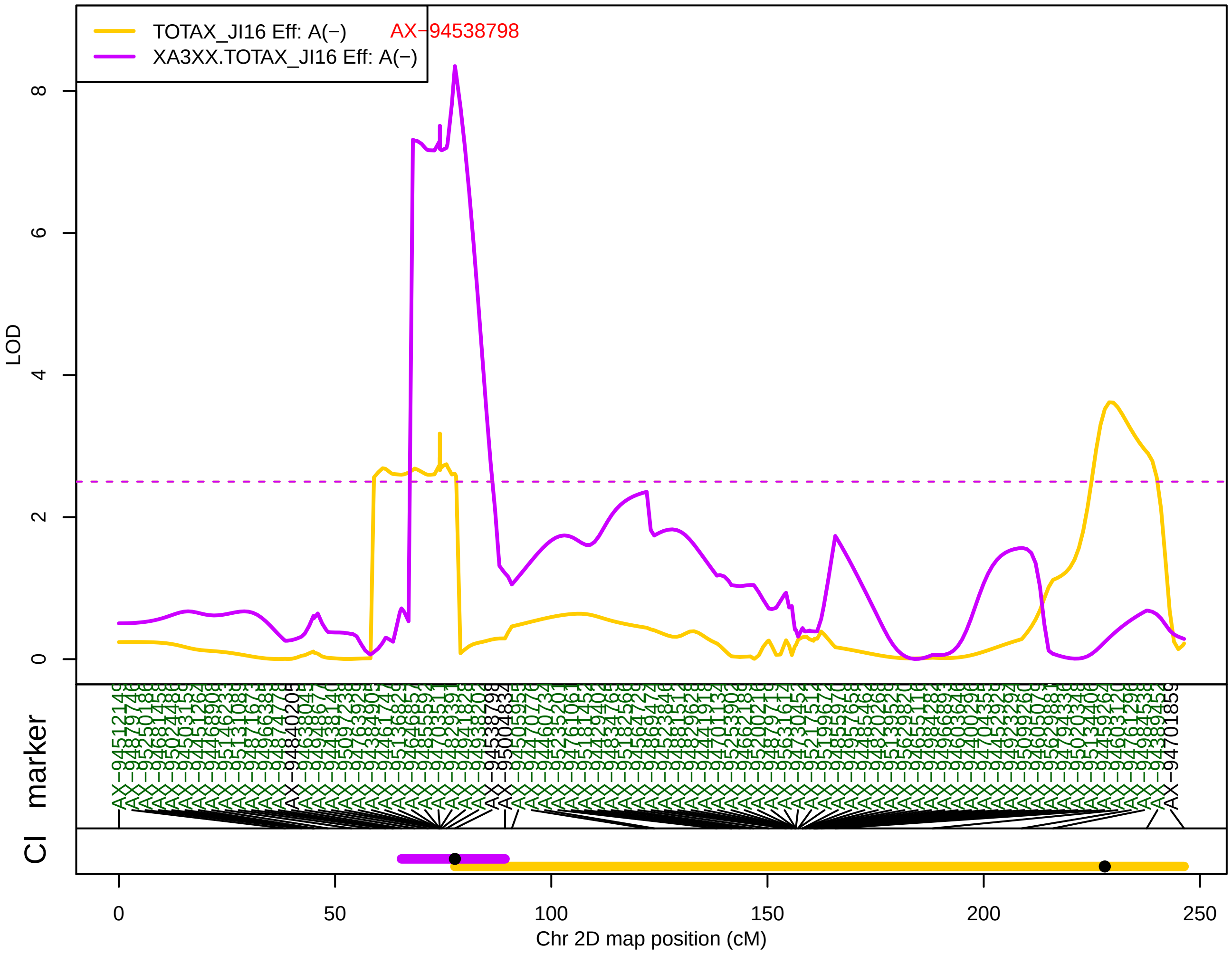

QTL AX−94389452|AX−94538798|AX−94389452|AX−94538798 (2018−08−01)

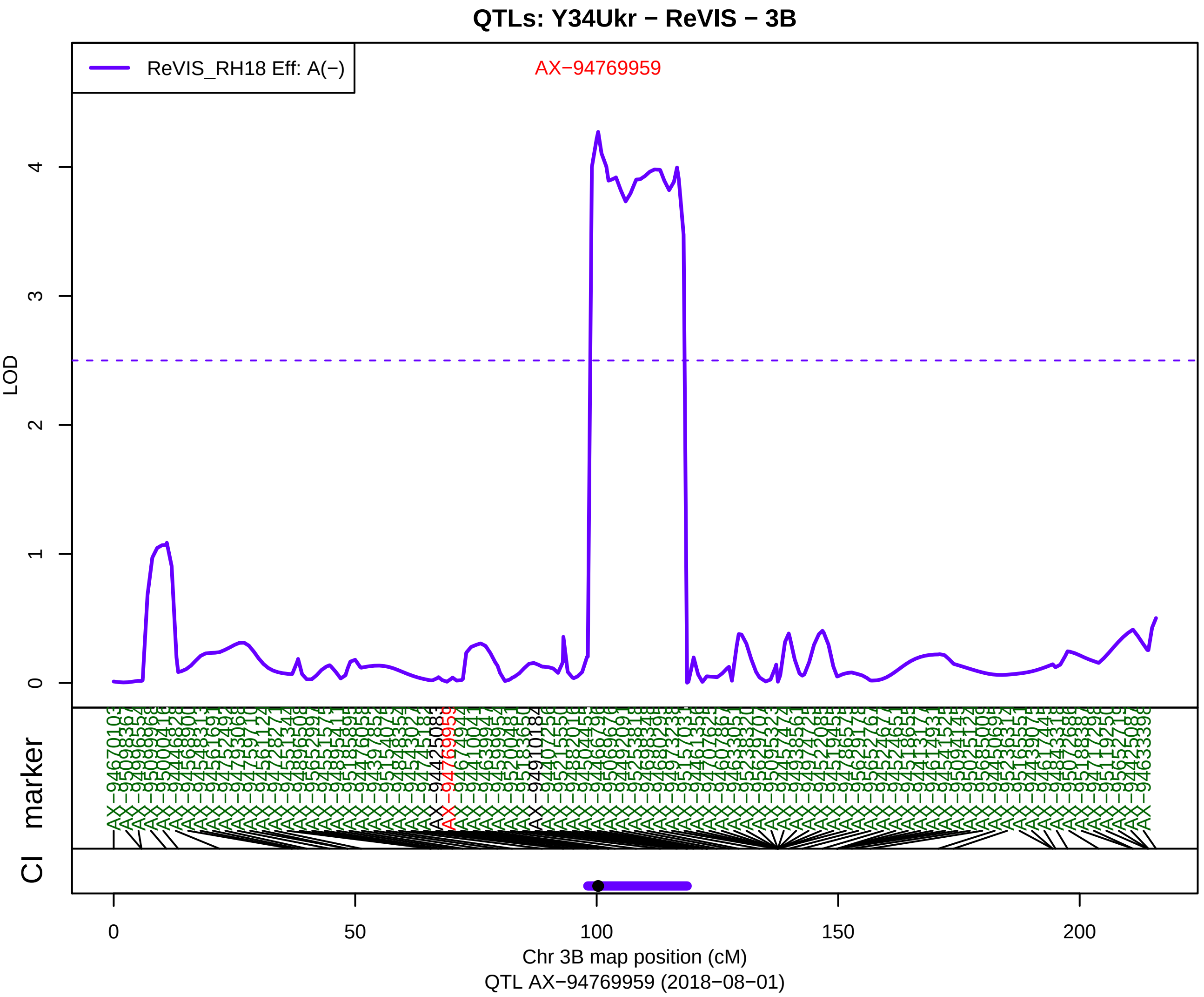

QTLs: Y34Ukr − ReVIS − 3B
ReVIS_RH18 Eff: A(−)
AX−94769959
LOD
0
1
2
3
4
marker
CI
0
50
100
150
200
Chr 3B map position (cM)
QTL AX−94769959 (2018−08−01)

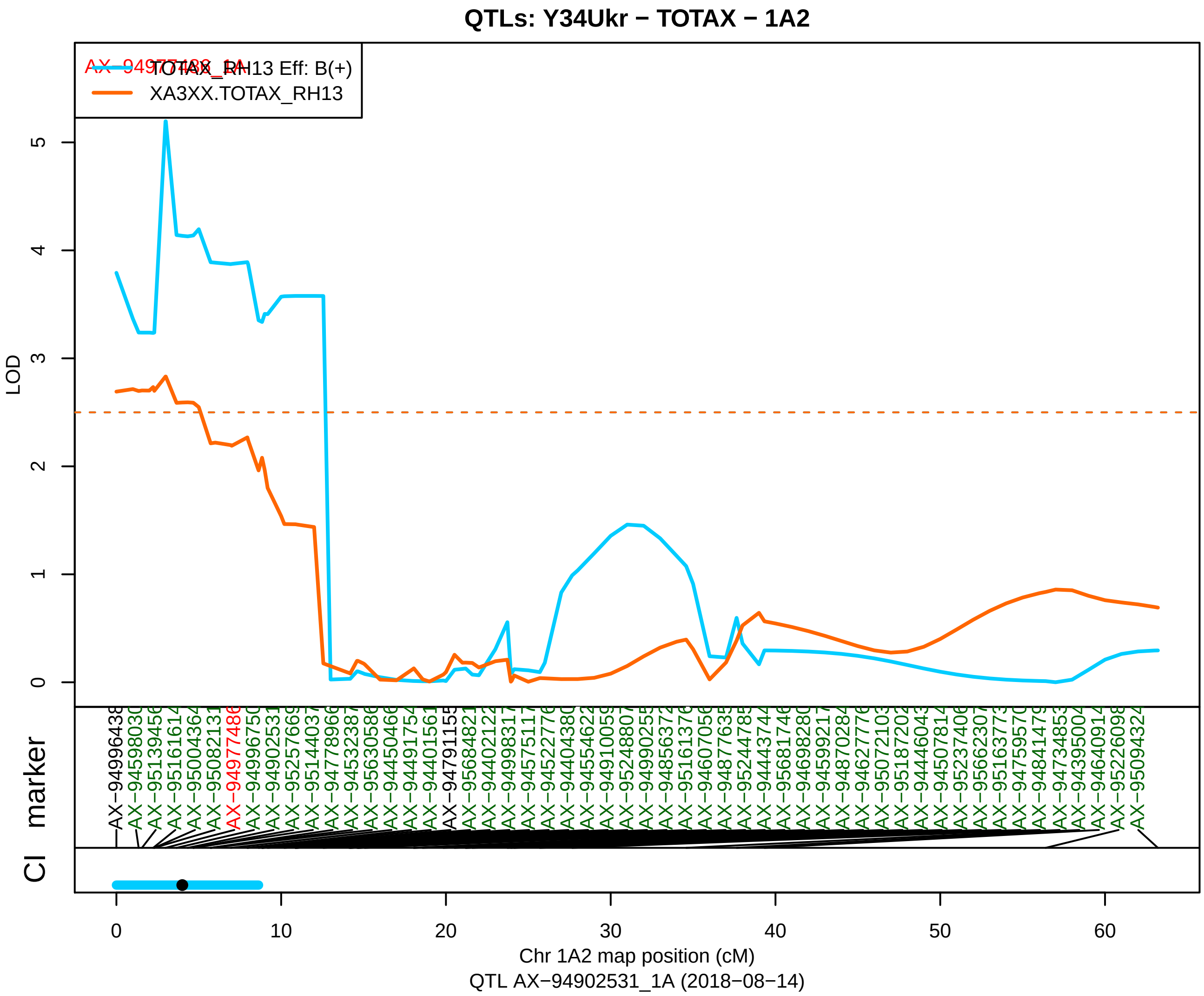
QTLs: Y34Ukr − TOTAX − 1A2
TOTAX_RH13 Eff: B(+)
AX−94977486_1A
XA3XX.TOTAX_RH13
LOD
0
1
2
3
4
5
marker
CI
AX−94996438
AX−94598030
AX−95139456
AX−95161614
AX−95004364
AX−95082131
AX−94977486
AX−94996750
AX−94902531
AX−95257669
AX−95144037
AX−94778966
AX−94532387
AX−95630586
AX−94450466
AX−94491754
AX−94401561
AX−94791155
AX−95684821
AX−94402122
AX−94998317
AX−94575117
AX−94522776
AX−94404380
AX−94554622
AX−94910059
AX−95248807
AX−94990255
AX−94856372
AX−95161376
AX−94607056
AX−94877635
AX−95244785
AX−94443744
AX−95681746
AX−94698280
AX−94599217
AX−94870284
AX−94627776
AX−95072103
AX−95187202
AX−94446043
AX−94507814
AX−95237406
AX−95662307
AX−95163773
AX−94759570
AX−94841479
AX−94734853
AX−94395004
AX−94640914
AX−95226098
AX−95094324
0
10
20
30
40
50
60
Chr 1A2 map position (cM)
QTL AX−94902531_1A (2018−08−14)

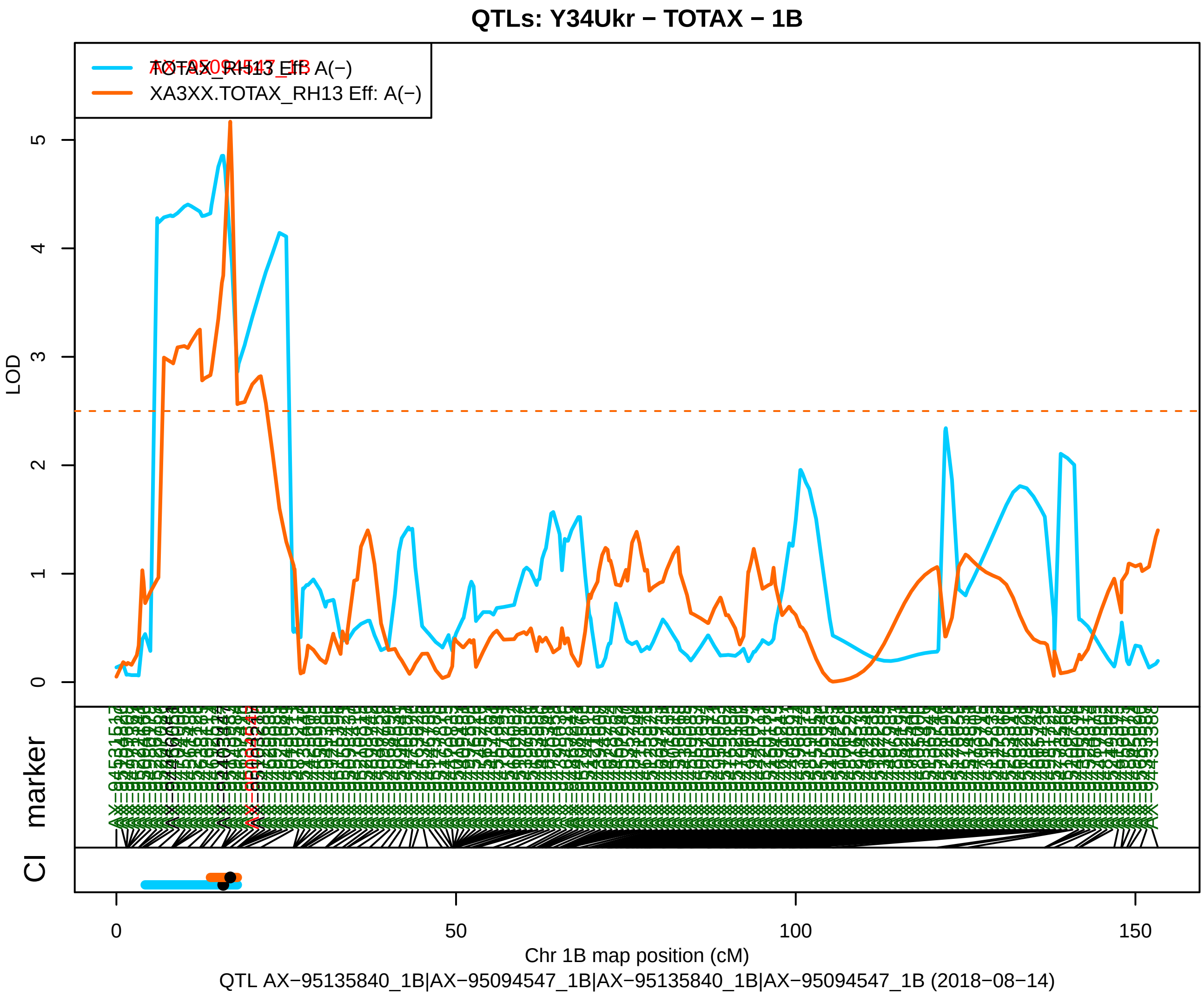
QTLs: Y34Ukr − TOTAX − 1B
TOTAX_RH13 Eff: A(−)
AX−95094547_1B
XA3XX.TOTAX_RH13 Eff: A(−)
LOD
0
1
2
3
4
5
marker
CI
0
50
100
150
Chr 1B map position (cM)
QTL AX−95135840_1B|AX−95094547_1B|AX−95135840_1B|AX−95094547_1B (2018−08−14)

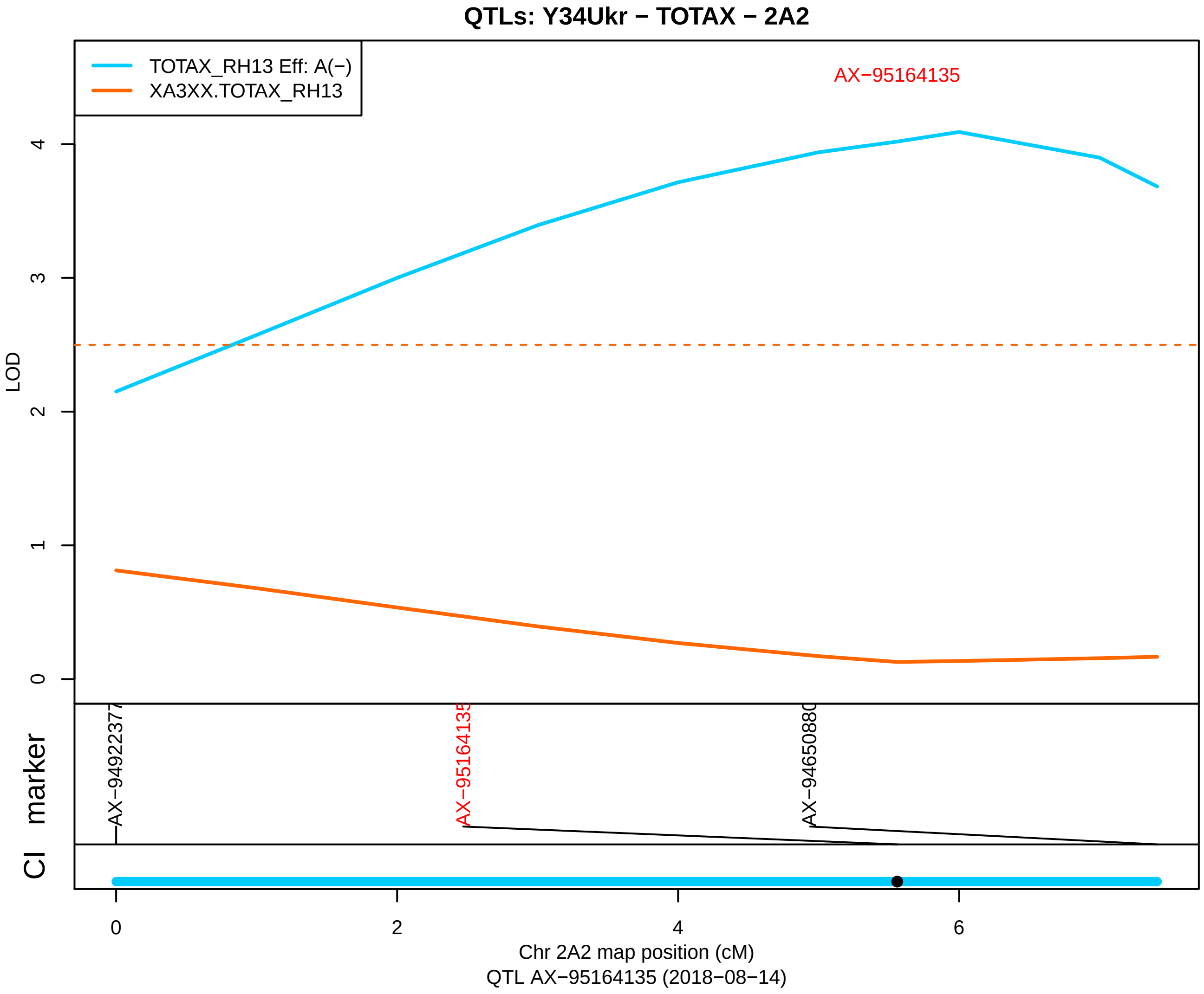

QTLs: Y34Ukr − TOTAX − 2A2
TOTAX_RH13 Eff: A(−)
XA3XX.TOTAX_RH13
AX−95164135
LOD
0
1
2
3
4
marker
CI
AX−94922377
AX−95164135
AX−94650880
0
2
4
6
Chr 2A2 map position (cM)
QTL AX−95164135 (2018−08−14)

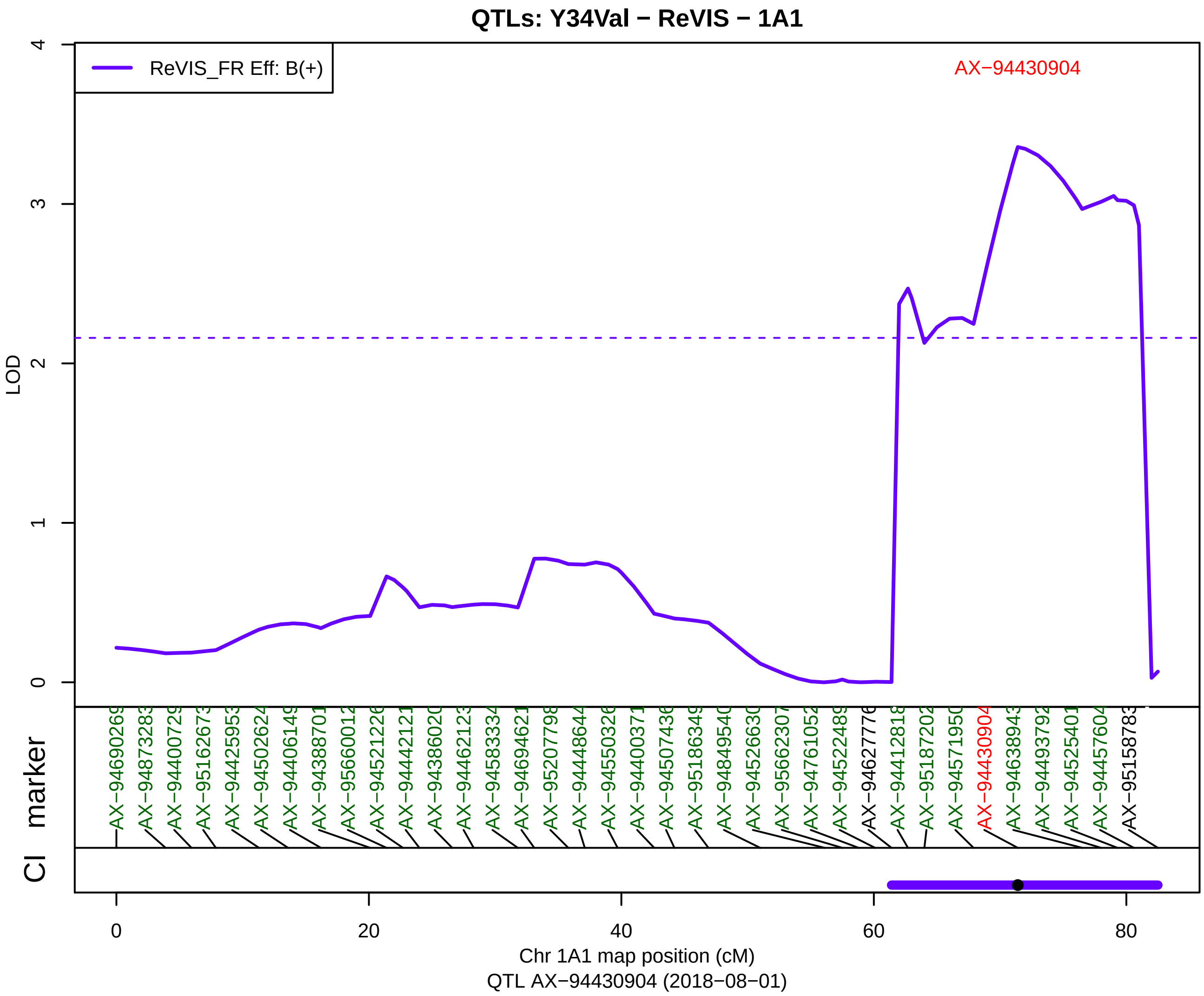
QTLs: Y34Val − ReVIS − 1A1
ReVIS_FR Eff: B(+)
AX−94430904
LOD
0
1
2
3
4
marker
CI
AX−94690269
AX−94873283
AX−94400729
AX−95162673
AX−94425953
AX−94502624
AX−94406149
AX−94388701
AX−95660012
AX−94521226
AX−94442121
AX−94386020
AX−94462123
AX−94583334
AX−94694621
AX−95207798
AX−94448644
AX−94550326
AX−94400371
AX−94507436
AX−95186349
AX−94849540
AX−94526630
AX−95662307
AX−94761052
AX−94522489
AX−94627776
AX−94412818
AX−95187202
AX−94571950
AX−94430904
AX−94638943
AX−94493792
AX−94525401
AX−94457604
AX−95158783
0
20
40
60
80
Chr 1A1 map position (cM)
QTL AX−94430904 (2018−08−01)

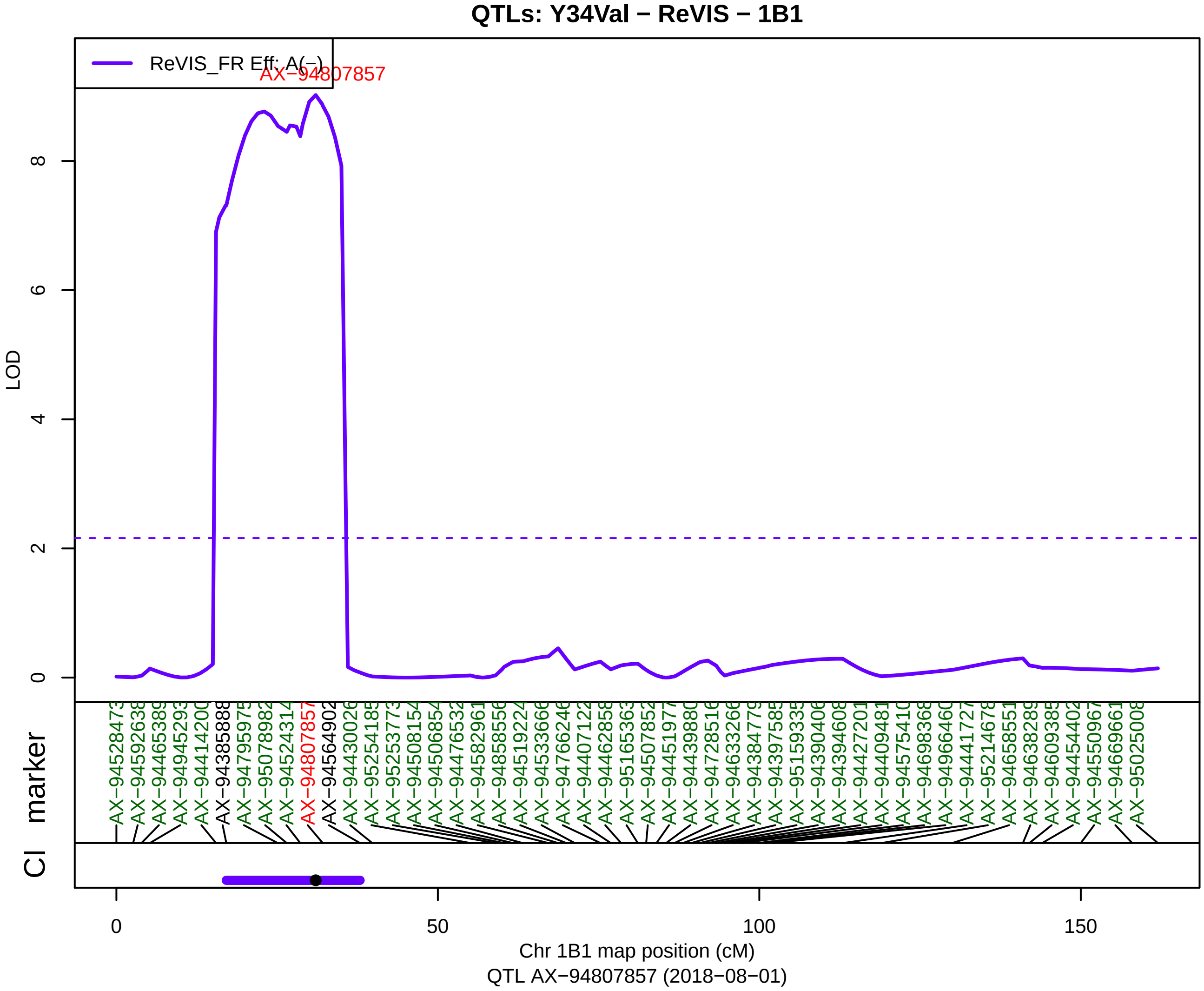
QTLs: Y34Val − ReVIS − 1B1
ReVIS_FR Eff: A(−)
AX−94807857
LOD
0
2
4
6
8
marker
CI
AX−94528473
AX−94592638
AX−94465389
AX−94945293
AX−94414200
AX−94385888
AX−94795975
AX−95078982
AX−94524314
AX−94807857
AX−94564902
AX−94430026
AX−95254185
AX−95253773
AX−94508154
AX−94506854
AX−94476532
AX−94582961
AX−94858556
AX−94519222
AX−94533666
AX−94766240
AX−94407122
AX−94462858
AX−95165363
AX−94507852
AX−94451977
AX−94439880
AX−94728516
AX−94633266
AX−94384779
AX−94397585
AX−95159335
AX−94390406
AX−94394608
AX−94427201
AX−94409481
AX−94575410
AX−94698368
AX−94966460
AX−94441727
AX−95214678
AX−94658551
AX−94638289
AX−94609385
AX−94454402
AX−94550967
AX−94669661
AX−95025008
0
50
100
150
Chr 1B1 map position (cM)
QTL AX−94807857 (2018−08−01)

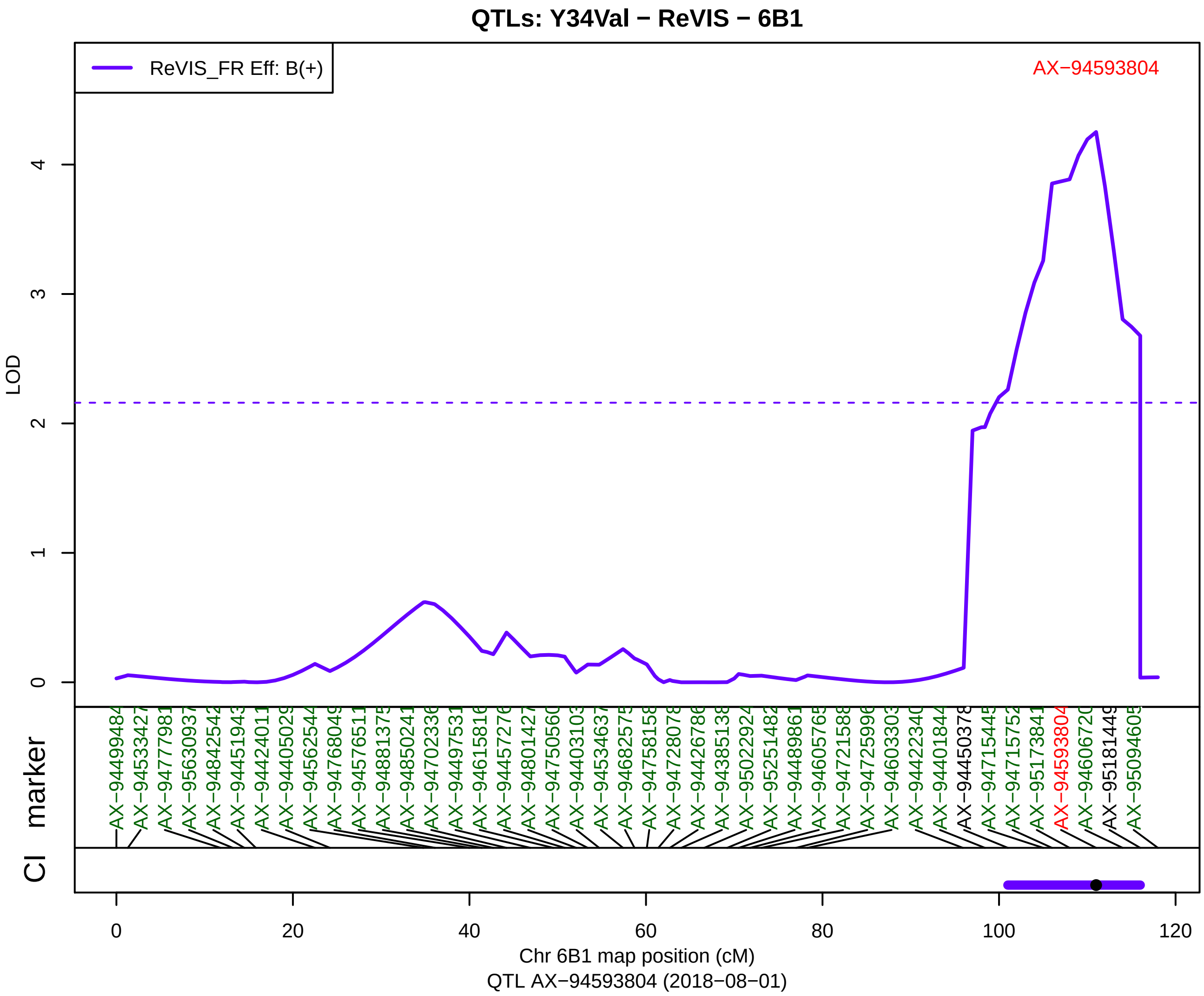
QTLs: Y34Val − ReVIS − 6B1
ReVIS_FR Eff: B(+)
AX−94593804
LOD
0
1
2
3
4
marker
CI
AX−94499484
AX−94533427
AX−94777981
AX−95630937
AX−94842542
AX−94451943
AX−94424011
AX−94405029
AX−94562544
AX−94768049
AX−94576511
AX−94881375
AX−94850241
AX−94702336
AX−94497531
AX−94615816
AX−94457276
AX−94801427
AX−94750560
AX−94403103
AX−94534637
AX−94682575
AX−94758158
AX−94728078
AX−94426786
AX−94385138
AX−95022924
AX−95251482
AX−94489861
AX−94605765
AX−94721588
AX−94725996
AX−94603303
AX−94422340
AX−94401844
AX−94450378
AX−94715445
AX−94715752
AX−95173841
AX−94593804
AX−94606720
AX−95181449
AX−95094605
0
20
40
60
80
100
120
Chr 6B1 map position (cM)
QTL AX−94593804 (2018−08−01)

QTLs: Y34Val − TOTAX − 1A1

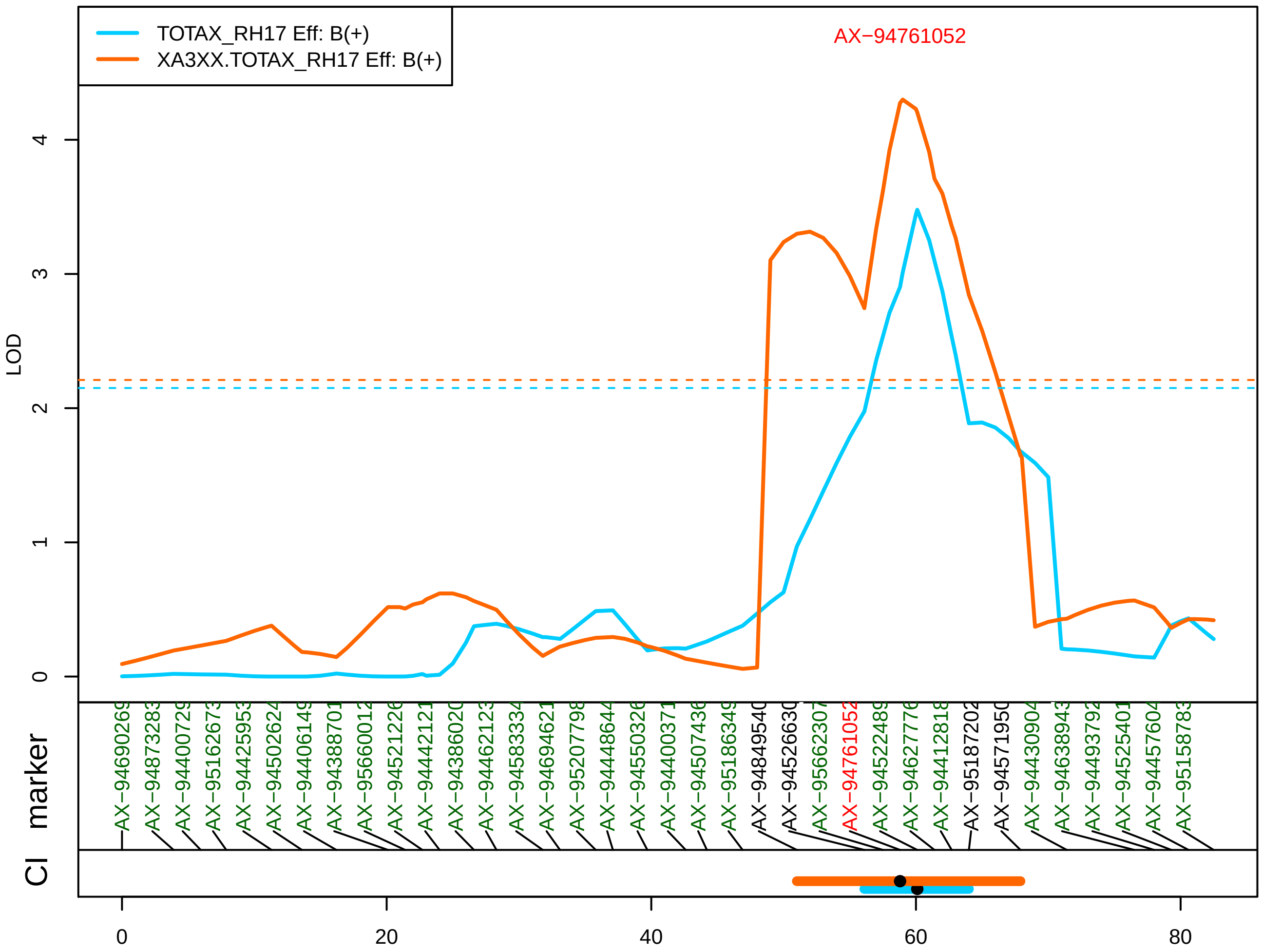

Chr 1A1 map position (cM)
QTL AX−94522489|AX−94761052|AX−94522489|AX−94761052 (2018−08−01)

QTLs: Y34Val − TOTAX − 1B1

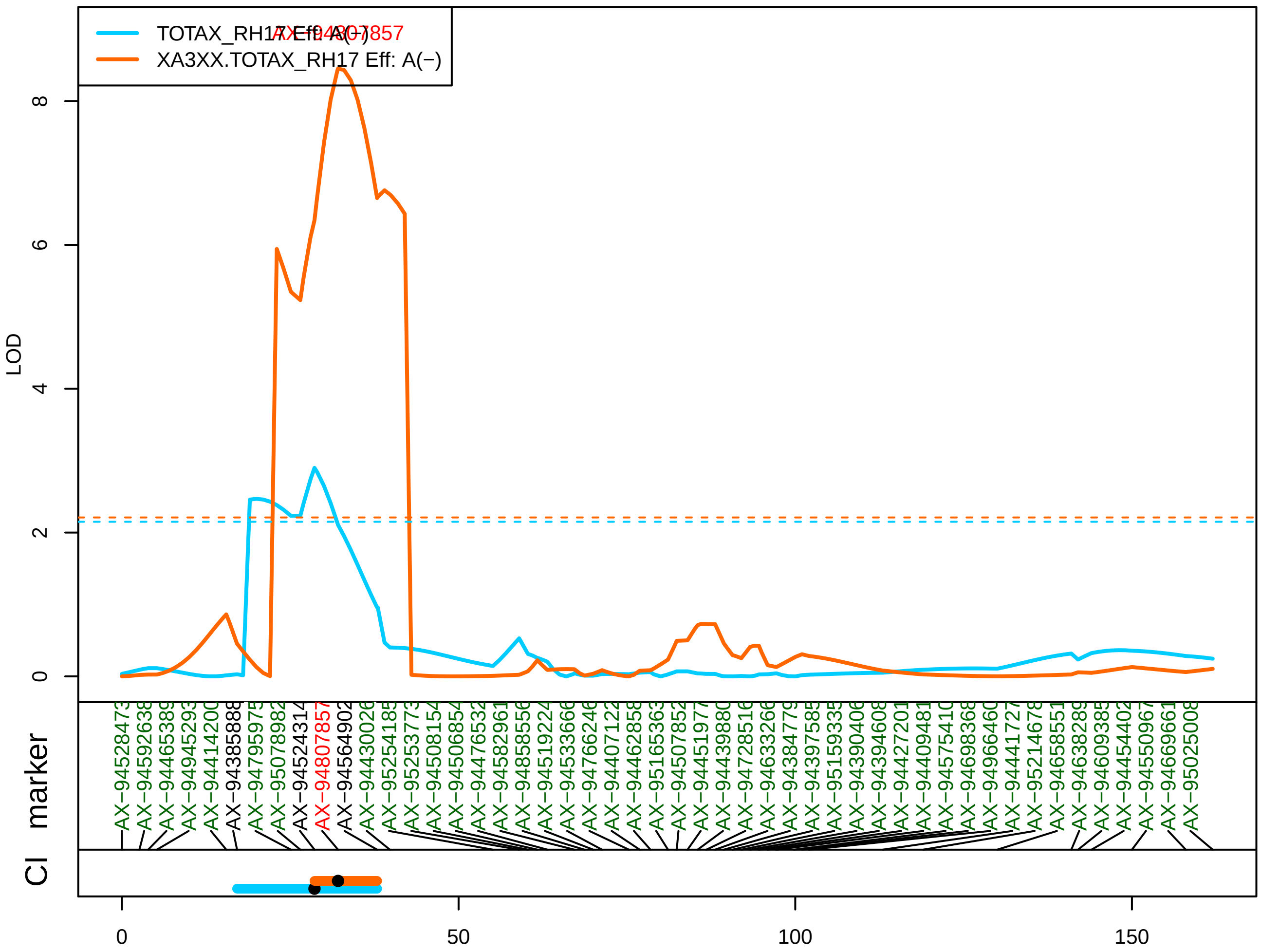

Chr 1B1 map position (cM)
QTL AX−94524314|AX−94807857|AX−94524314|AX−94807857 (2018−08−01)
