# Supplementary material for: Identification of a major QTL and associated molecular marker for high arabinoxylan fibre in white wheat flour
Source: PLoS One. 2020 Feb 5;15(2):e0227826. doi: 10.1371/journal.pone.0227826 (PMC7001892; doi:10.1371/journal.pone.0227826)
Supplement: S3 Fig — WE-AX and TOT-AX were measured colorometrically (pentosan assay) and TOT-AX by enzyme fingerprinting in wholemeal and white flour. (PDF) [file pone.0227826.s003.pdf]

	White WE (pentosans)	White Total (pentosans)	White Total AX (fingerprinting)	White WE (relative viscosity)	Wholemeal WE (pentosans)	Wholemeal Total (pentosans)	Wholemeal Total AX (fingerprinting)
**White Total (pentosans)**	0.62						
**White Total AX (fingerprinting)**	0.65	0.67					
**White WE (relative viscosity)**	0.62	0.52	0.75				
**Wholemeal WE (pentosans)**	0.96	0.52	0.59	0.5			
**Wholemeal Total (pentosans)**	0.18	0.23	-0.24	-0.25	0.22		
**Wholemeal Total AX (fingerprinting)**	0.81	0.76	0.83	*0.63*	0.74	0.05	
**Wholemeal WE (relative viscosity)**	**0.77**	0.38	0.55	**0.84**	**0.7**	-0.03	0.63

S3 Figure. Correlation Matrix of 10 lines from the Yumai x Ukrainka population grown at Rothamsted Research in 2013-2014.

Relative viscosity, water extractable (WE) and total AX measured colorometrically (pentosan assay), and total AX by enzyme fingerprinting in wholemeal and white flour..
