# Supplementary material for: Identification of a major QTL and associated molecular marker for high arabinoxylan fibre in white wheat flour
Source: PLoS One. 2020 Feb 5;15(2):e0227826. doi: 10.1371/journal.pone.0227826 (PMC7001892; doi:10.1371/journal.pone.0227826)
Supplement: S1 Table — Abbreviations: Y34 Yumai 34; Alt Altigo; Cla Claire; Ukr Ukrainka; Val Valoris; chr chromosome on which QTL were identified; env: environment (location and year): FR: Clermont Ferrand, JI: John Innes Centre, RH: Rothamsted Research; %var percentage of phenotypic variance explained; increasAll: increasing allele (A = Y34, B = second parent); LG chr: linkage group on the genetic map; cM: centiMorgan; CI confidence interval of the QTL; RefSeq1.0 Chr: chromosome on the IWGCS Reference Sequence (vs 1.0) whole genome assembly; pos: positions on the chromosome, if given in base pairs (bp) positions refer to RefSeq1.0. (PDF) [file pone.0227826.s004.pdf]

Major QTL

population	Chr	env	trait	LOD	%var	add eff	phenotype mean	increase All	LG chr	QTL peak pos [cM]	pos nearest marker	QTL peak, nearest marker	CI start pos [cM]	CI start marker	CI end pos [cM]	CI end marker	RefSeq 1.0 Chr	QTL peak pos [bp]	CI start pos [bp]	CI end pos [bp]
Y34Alt	1B	FR13	RV	12.6	16.3	-0.53	3	A	1B	26	26	AX-94618000	25.6	AX-94564902	27.5	AX-94385888	1B	649369083	646172163	660529537
Y34Alt	2D	FR13	RV	3.2	3.7	-0.26	3	A	2D1	9.5	9.5	AX-94452103	5.9	AX-94860457	14.3	AX-94568326	2D		156825008	599946636
Y34Alt	3B	FR13	RV	6.1	7.4	-0.3	3	A	3B1	34.2	34.2	AX-94382595	31.4	AX-94480990	35.8	AX-94624133	3B	512742728	417261367	512742728
Y34Alt	4D	FR13	RV	3.5	4.1	-0.27	3	A	4D1	1	0.9	AX-94766682	0	AX-94917034	4.1	AX-94394903	4D	426519545	31652113	426519545
Y34Cla	1B	JI15	TOT-AX	3.2	14.1	-3.8	74.4	A	1B	38	35.9	AX-94385888	23.6	AX-94521944	45	AX-94510581	1B	660529537	656658155	668056547
Y34Cla	2B	JI16	RV	3.1	11.4	-0.04	1.8	A	2B	158	159	AX-94421649	0	AX-94648255	268.2	AX-94529349	2B	249418689	4503734	747573949
Y34Cla	3B	JI16	RV	5.9	23.9	-0.01	1.8	A	3B	148	150.3	AX-95629178	145.6	AX-95259479	154.9	AX-95094145	3B	488873234	474767686	522395687
Y34Ukr	1A	RH13	TOT-AX	3.2	13	2.1	95.1	B	1A2	11.5	11.5	AX-94902531	4.3	AX-94996438	17.5	AX-95684821	1A	8130190	1145413	12157282
Y34Ukr	1B	RH13	TOT-AX	5.1	21.6	-3	95.1	A	1B	227.7	227.7	AX-94845742	219.3	AX-95094547	241.9	AX-95222141	1B	15745889	1252520	23978766
Y34Val	1A	FR13	RV	3.8	10.3	0.62	4.5	B	1A1	71.4	71.4	AX-94430904	61.4	AX-94627776	82.5	AX-95158783	1A	21282281	3845868	30193309
Y34Val	1B	FR13	RV	7.8	24.2	-0.99	4.5	A	1B1	31	32.1	AX-94807857	17.1	AX-94385888	37.9	AX-94564902	1B	652575871	646172163	660529537
Y34Val	6B	FR13	RV	4.4	12.3	0.85	4.5	B	6B1	111	111	AX-94593804	101	AX-94450378	116	AX-95181449	6B	22233686	11299395	28116150

Minor QTL

population	Chr	env	trait	LOD	%var	add eff	phenotype mean	increase All	LG chr	QTL peak pos [cM]	pos nearest marker	QTL peak, nearest marker	CI start pos [cM]	CI start marker	CI end pos [cM]	CI end marker	RefSeq 1.0 Chr	QTL peak pos [bp]	CI start pos [bp]	CI end pos [bp]
Y34Alt	2B	FR13	RV	2.7	3.1	-0.26	3	A	2B	44.9	44.9	AX-94546045	42.2	AX-94428130	47	AX-95171852	2B	47425660	45787181	75613709
Y34Alt	4B	FR13	RV	2.4	2.9	0.22	3	B	4B1	4.8	4.8	AX-94853726	0.4	AX-94924826	6.8	AX-94615340	4B	17270078	6030086	31707105
Y34Cla	2D	JI16	TOT-AX	2.5	12.4	1.76	8	A	2D	228	237.7	AX-94389452	77.7	AX-94538798	246.3	AX-94701859	2D	6903817	6903817	601245173
Y34Cla	3A	JI16	RV	2	7.3	0.02	1.8	B	3A1	67.3	67.3	AX-94603083	59	AX-95194580	86.3	AX-94833103	3A	602680124	485011990	613393005
Y34Cla	5D	JI15	TOT-AX	1.6	7	-0.08	74.4	B	5D2	62	59.7	AX-94877826	26.6	AX-94422079	76.5	AX-94391831	5D	453240756	97827010	439786302
Y34Ukr	2A	RH13	TOT-AX	2.9	9.7	0.04	95.1	A	2A2	5.6	5.6	AX-95164135	0	AX-94922377	7.4	AX-94650880	2A	5400000	2151813	8719514
Y34Ukr	3B	RH13	RV	2.9	14.3	-0.06	1.8	B	3B	100.3	100.3	AX-94769959	98.2	AX-94425083	118.7	AX-94910184	3B	190889006	167734656	452103409
Y34Val	1A	FR13	TOT-AX	2.4	12	1.16	10.7	B	1A1	60.1	60.1	AX-94522489	56.1	AX-94526630	64	AX-95187202	1A	32302672	24944673	42550052
Y34Val	1B	FR13	TOT-AX	2.5	12.6	-1.18	10.7	A	1B1	28.6	28.6	AX-94524314	17.1	AX-94385888	37.9	AX-94564902	1B	656852312	646172163	660529537
