# Supplementary material for: Identification of a major QTL and associated molecular marker for high arabinoxylan fibre in white wheat flour
Source: PLoS One. 2020 Feb 5;15(2):e0227826. doi: 10.1371/journal.pone.0227826 (PMC7001892; doi:10.1371/journal.pone.0227826)
Supplement: S2 Table — (PDF) [file pone.0227826.s005.pdf]

S2 Table. Comparison of the contents of total and water-extractable AX (determined as pentosan) in white flour from three wheat cultivars grown in field trials over 10 years (2009-2018).

Cultivar		Total pentosan (mg/ g dry weight)	Water-extractable pentosan (mg/ g dry weight)
**Yumai 34**	range	18.60-23.38	8.43-11.41
	mean	20.65	9.52
	SD	1.76	0.87
**Ukrainka**	range	14.29-18.19	4.35-6.93
	mean	16.45	5.68
	SD	1.48	0.83
**Lupus**	range	14.06-18.76	5.42-7.28
	mean	16.48	6.52
	SD	1.81	0.64
