# Supplementary material for: Identification of a major QTL and associated molecular marker for high arabinoxylan fibre in white wheat flour
Source: PLoS One. 2020 Feb 5;15(2):e0227826. doi: 10.1371/journal.pone.0227826 (PMC7001892; doi:10.1371/journal.pone.0227826)
Supplement: S3 Table — (PDF) [file pone.0227826.s006.pdf]

S3 Table. Marker trait associations identified for relative viscosity in the WHEALBI panel.

marker	chrom	position	VP (-LogPvalue)	REF	ALT	Closest HC	Closest LC
WTa_011962		652455501	1.19E+01	T	C	TraesCS1B01G426300	TraesCS1B01G723400LC
WTa_09ac26		652302035	1.16E+01	T	C	TraesCS1B01G426000	TraesCS1B01G723300LC
WTa_09ac27	1B	652302058	1.16E+01	A	C	TraesCS1B01G426000	TraesCS1B01G723300LC
WTa_09ac28		652302152	1.16E+01	G	A	TraesCS1B01G426000	TraesCS1B01G723300LC
WTa_011959		652453983	1.12E+01	G	A	TraesCS1B01G426300	TraesCS1B01G723400LC
WTa_074b4f		26076251	1.01E+01	A	T	TraesCS6B01G042500	TraesCS6B01G056200LC
WTa_074b50		26076566	1.01E+01	G	C	TraesCS6B01G042600	TraesCS6B01G056200LC
WTa_074b53	6B	26076692	1.01E+01	C	T	TraesCS6B01G042600	TraesCS6B01G056200LC
WTa_074b54		26076727	1.01E+01	C	T	TraesCS6B01G042600	TraesCS6B01G056200LC
WTa_0b5393		26077228	1.01E+01	C	T	TraesCS6B01G042600	TraesCS6B01G056200LC

marker	chrom	position	VP (-LogPvalue)	REF	ALT	Closest HC	Closest LC
WTa_011962		652455501	1.19E+01	T	C	TraesCS1B01G426300	TraesCS1B01G723400LC
WTa_09ac26		652302035	1.16E+01	T	C	TraesCS1B01G426000	TraesCS1B01G723300LC
WTa_09ac27	1B	652302058	1.16E+01	A	C	TraesCS1B01G426000	TraesCS1B01G723300LC
WTa_09ac28		652302152	1.16E+01	G	A	TraesCS1B01G426000	TraesCS1B01G723300LC
WTa_011959		652453983	1.12E+01	G	A	TraesCS1B01G426300	TraesCS1B01G723400LC
WTa_074b4f	6B	26076251	1.01E+01	A	T	TraesCS6B01G042500	TraesCS6B01G056200LC

WTa_074b50		26076566	1.01E+01	G	C	TraesCS6B01G042600	TraesCS6B01G056200LC
WTa_074b53		26076692	1.01E+01	C	T	TraesCS6B01G042600	TraesCS6B01G056200LC
WTa_074b54		26076727	1.01E+01	C	T	TraesCS6B01G042600	TraesCS6B01G056200LC
WTa_0b5393		26077228	1.01E+01	C	T	TraesCS6B01G042600	TraesCS6B01G056200LC

marker	chrom	position	VP (-LogPvalue)	REF	ALT	Closest HC	Closest LC
WTa_011962		652455501	1.19E+01	T	C	TraesCS1B01G426300	TraesCS1B01G723400LC
WTa_09ac26		652302035	1.16E+01	T	C	TraesCS1B01G426000	TraesCS1B01G723300LC
WTa_09ac27	1B	652302058	1.16E+01	A	C	TraesCS1B01G426000	TraesCS1B01G723300LC
WTa_09ac28		652302152	1.16E+01	G	A	TraesCS1B01G426000	TraesCS1B01G723300LC
WTa_011959		652453983	1.12E+01	G	A	TraesCS1B01G426300	TraesCS1B01G723400LC
WTa_074b4f		26076251	1.01E+01	A	T	TraesCS6B01G042500	TraesCS6B01G056200LC
WTa_074b50		26076566	1.01E+01	G	C	TraesCS6B01G042600	TraesCS6B01G056200LC
WTa_074b53	6B	26076692	1.01E+01	C	T	TraesCS6B01G042600	TraesCS6B01G056200LC
WTa_074b54		26076727	1.01E+01	C	T	TraesCS6B01G042600	TraesCS6B01G056200LC
WTa_0b5393		26077228	1.01E+01	C	T	TraesCS6B01G042600	TraesCS6B01G056200LC

Markers as in Pont et al. [32]; chrom is chromosome alignment of marker position to the Chinese Spring reference genome and position in bp; LogP is the log p value of the association, REF is the base at the marker position in the Chinese Spring reference genome; ALT is the alternative allele present in the panel; Closest HC and LC are the nearest high and low confidence gene models, respectively.
